# Cations and Anions of Dibenzo[*a*,*e*]pentalene and Reduction of a Dibenzo[*a*,*e*]pentalenophane

**DOI:** 10.1002/chem.202005131

**Published:** 2021-02-12

**Authors:** Mathias Hermann, Tobias Böttcher, Marcel Schorpp, Sabine Richert, Daniel Wassy, Ingo Krossing, Birgit Esser

**Affiliations:** ^1^ Institute for Organic Chemistry University of Freiburg Albertstraße 21 79104 Freiburg Germany; ^2^ Institute for Inorganic and Analytical Chemistry University of Freiburg Albertstraße 21 79104 Freiburg Germany; ^3^ Institute of Physical Chemistry University of Freiburg Albertstraße 21 79104 Freiburg Germany; ^4^ Freiburg Materials Research Center University of Freiburg Stefan-Meier-Str. 21 79104 Freiburg Germany; ^5^ Freiburg Center for Interactive Materials and Bioinspired Technologies University of Freiburg Georges-Köhler-Allee 105 79110 Freiburg Germany

**Keywords:** antiaromaticity, organic field-effect transistors, oxidation, radical anion, reduction

## Abstract

Dibenzo[*a*,*e*]pentalene (DBP) is a non‐alternant conjugated hydrocarbon with antiaromatic character and ambipolar electrochemical behavior. Upon both reduction and oxidation, it becomes aromatic. We herein study the chemical oxidation and reduction of a planar DBP derivative and a bent DBP‐phane. The molecular structures of its planar dication, cation radical and anion radical in the solid state demonstrate the gained aromaticity through bond length equalization, which is supported by nucleus independent chemical shift‐calculations. EPR spectra on the cation radical confirm the spin delocalization over the DBP framework. A similar delocalization was not possible in the reduced bent DBP‐phane, which stabilized itself by proton abstraction from a solvent molecule upon reduction. This is the first report on structures of a DBP cation radical and dication in the solid state and of a reduced bent DBP derivative. Our study provides valuable insight into the charged species of DBP for its application as semiconductor.

## Graphical abstract

**Aromatic character** in a dibenzo[*a*,*e*]pentalene (DBP) was observed upon chemical oxidation to the cation radical and dication and reduction to the anion radical, as found from solid‐state structures. In a bent DBP‐phane aromaticity could not be reached upon reduction, and stabilization though proton abstraction occurred.

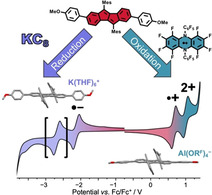

## Introduction

**Figure 1:**
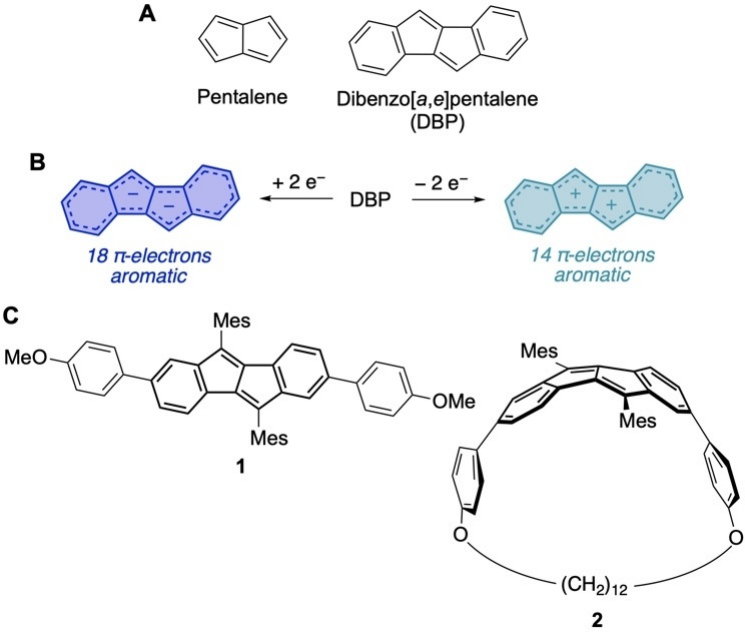
A) Molecular structures of pentalene and dibenzo[*a*,*e*]pentalene (DBP); B) The twofold oxidation or reduction of DBP leads to an aromatic dication or dianion, respectively; C) DBP‐derivative **1** and (2,7)dibenzo[*a*,*e*]pentalenophane **2** used in this study.

Polycyclic aromatic hydrocarbons (PAHs) incorporating five‐membered rings have intrigued chemists for decades. As opposed to alternant PAHs composed of only six‐membered rings, those with five‐membered rings are non‐alternant π‐systems.^[1]^ The result is a shift of orbital energies relative to an alternant π‐system of similar size, in most cases an increase in HOMO and a decrease in LUMO energy. This bestows ambipolar electrochemical character and a small band gap on five‐membered ring‐containing PAHs. Furthermore, they can possess antiaromatic character.[^2^, ^3^, ^4^, ^5^, ^6^, ^7^] The best example here is pentalene (Figure 1 A), which is so unstable and reactive that it could only be isolated when bearing three kinetically stabilizing *tert*‐butyl substituents.^[8]^

Another strategy of stabilization is to annulate two benzene rings to the pentalene core, resulting in dibenzo[*a*,*e*]pentalene (DBP, Figure 1 A),^[9]^ which is a thermodynamically and kinetically stable compound.^[10]^ Yet it retains a small band gap, reflected in its red color, and ambipolar redox properties (Figure 1 B). The relative ease of oxidation and reduction of DBP can be explained by an increase in aromatic character. Formally, both two‐electron oxidation or ‐reduction render the 16‐π‐electron system Hückel‐aromatic with 14‐ or 18 π‐electrons, respectively (Figure 1 B). Not surprisingly, the interest in DBP's redox chemistry dates back to 1963 when Silvestri provided first experimental proof that a two‐fold reduction was possible.^[11]^ Rabinovitz[^12^, ^13^, ^14^] as well as Edlund^[15]^ and co‐workers followed with more detailed NMR‐ and EPR‐spectroscopic studies, confirming an increased aromatic character of the oxidized and reduced DBP. Finally, proof for the planar structure of the DBP dianion in the solid state, a further indication for its aromaticity, was obtained by Saito et al. in 2007.^[16]^ More examples of dianionic DBPs in the solid state followed[^17^, ^18^, ^19^] as well as the structure of a DBP anion radical.^[20]^ Similarly, the structures of the reduction products of π‐extended indenofluorenes were reported.[^21^, ^22^] However, no examples of a DBP cation radical or dication in the solid state have been reported yet.^[23]^

The oxidation and reduction products of DBP are also of interest regarding the application of DBPs as semiconductors in organic field‐effect transistors (OFETs). DBP derivatives and further benzannulated diaceno[*a*,*e*]pentalenes have been employed in both *p*‐[^24^, ^25^, ^26^, ^27^, ^28^, ^29^] and n‐type^[30]^ OFET devices. The charge transport is thought to proceed through either the cation radical in the case of *p*‐type doping or the anion radical for an n‐type device. Which type of doping is easier to achieve depends on the HOMO and LUMO energy levels of the DBP derivatives.[^31^, ^32^] Investigating charged DBP derivatives, which are responsible for charge carrier transport in devices, as discrete species gives insight into their electronic and geometric structures.

For these reasons we herein study the chemical oxidation and reduction of DBP derivative **1** (Figure 1 C).^[33]^ We were able to obtain its anion radical **1^.−^**, cation radical **1^.+^** and dication **1^2+^** and investigated their structural and electronic properties by X‐ray crystallography, EPR spectroscopy and DFT calculations. With the synthesis of (2,7)dibenzo[*a*,*e*]pentalenophanes^[33]^ (DBP‐phanes) and DBP‐based nanohoops[^34^, ^35^, ^36^] we recently showed that the DBP unit can be bent^[37]^ without strongly altering its optoelectronic properties. Hence, we herein performed a chemical reduction of DBP‐phane **2** (Figure 1 C) to investigate the influence of bending. We surprisingly found that the strain led to a reduction product different from that of planar **1** through proton abstraction from a solvent molecule.

## Results and Discussion

### Oxidation and reduction of planar DBP 1

**Figure 2:**
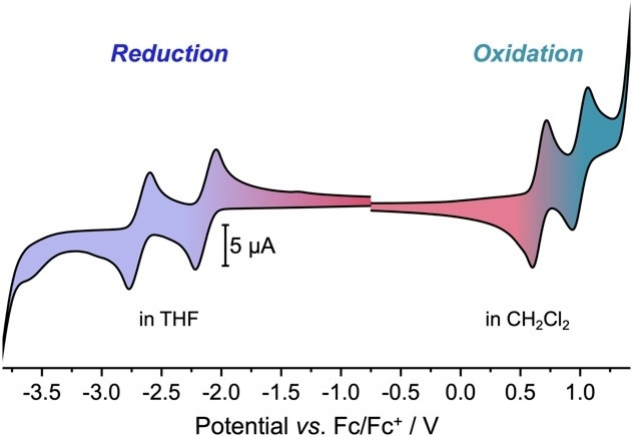
Cyclic voltammograms of DBP **1** in THF (reduction) and CH_2_Cl_2_ (oxidation) (1 mm, 0.1 m *n*Bu_4_NPF_6_, scan rate 0.1 V s^−1^, glassy carbon electrode, potentials given versus Fc/Fc^+^ as internal standard).

Planar DBP **1** was synthesized as previously described.^[33]^ Cyclic voltammetry measurements indicated that **1** could form both a relatively stable anion radical and dianion as well as cation radical and dication, testifying to its ambipolar electrochemical character (Figure 2). **1** featured two reversible reductions in THF with half‐wave potentials of *E*_1/2_=−2.13 and −2.69 V and two reversible oxidations in CH_2_Cl_2_ at *E*_1/2_=0.66 and 1.01 V (all vs. Fc/Fc^+^).

**Scheme 1:**
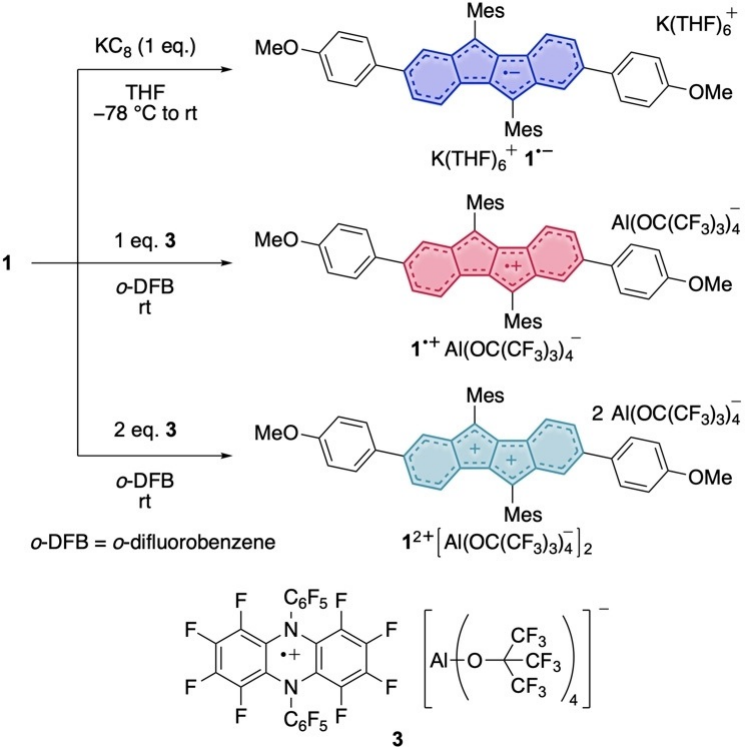
Synthesis of the DBP‐anion radical **1^.−^**, the cation radical **1^.+^** and the dication **1^2+^**.

For the chemical reduction of DBP **1**, one equivalent of KC_8_ was added to a solution of **1** in THF at −78 °C. After reaching room temperature, filtration afforded the anion radical as the salt [K(THF)_6_]^+^ **1^.−^** (Scheme 1) as an intensely dark‐blue‐colored solution. A concentrated solution of [K(THF)_6_]**1** in THF at −40 °C provided single crystals suitable for XRD. Attempts to obtain the dianion **1^2−^** by using two equivalents of KC_8_ were unsuccessful and only furnished the anion radical **1^.−^**. This may be due to the steric hindrance through the mesityl groups, which do not allow for an *η* ^5^‐coordination of the potassium ions to the reduced DBP core. In all reported solid‐state structure of DBP dianions in the literature with alkaline counter cations, both metal ions *η* ^5^‐coordinated to the DBP core.[^16^, ^18^, ^19^]

For the oxidation of **1** a strong yet innocent oxidant, facilitating an outer‐sphere electron transfer, was required. Based on its high oxidation potential (see above), we used the perfluorinated dihydrophenazine cation radical of salt **3**, which was recently introduced as a strong oxidant.^[38]^

Its formal reduction potential lies at 1.29 V versus Fc/Fc^+^ in *ortho*‐difluorobenzene (*o*‐DFB), hence sufficiently high to oxidize **1** to a dication. The large Al‐based weakly coordinating counterion Al(OR^F^)_4_]^−^ (R^F^=C(CF_3_)_3_)^[39]^ serves to stabilize generated reactive cationic species.[^40^, ^41^, ^42^, ^43^, ^44^] Indeed, treatment of **1** with one or two equivalents of **3** in *o*‐DFB furnished cation radical **1^.+^** and dication **1^2+^** as deeply red‐ and blue‐colored solutions, respectively. Layering of the obtained reaction mixtures with pentane afforded single crystals suitable for X‐ray diffraction analysis of both salts.

**Figure 3:**
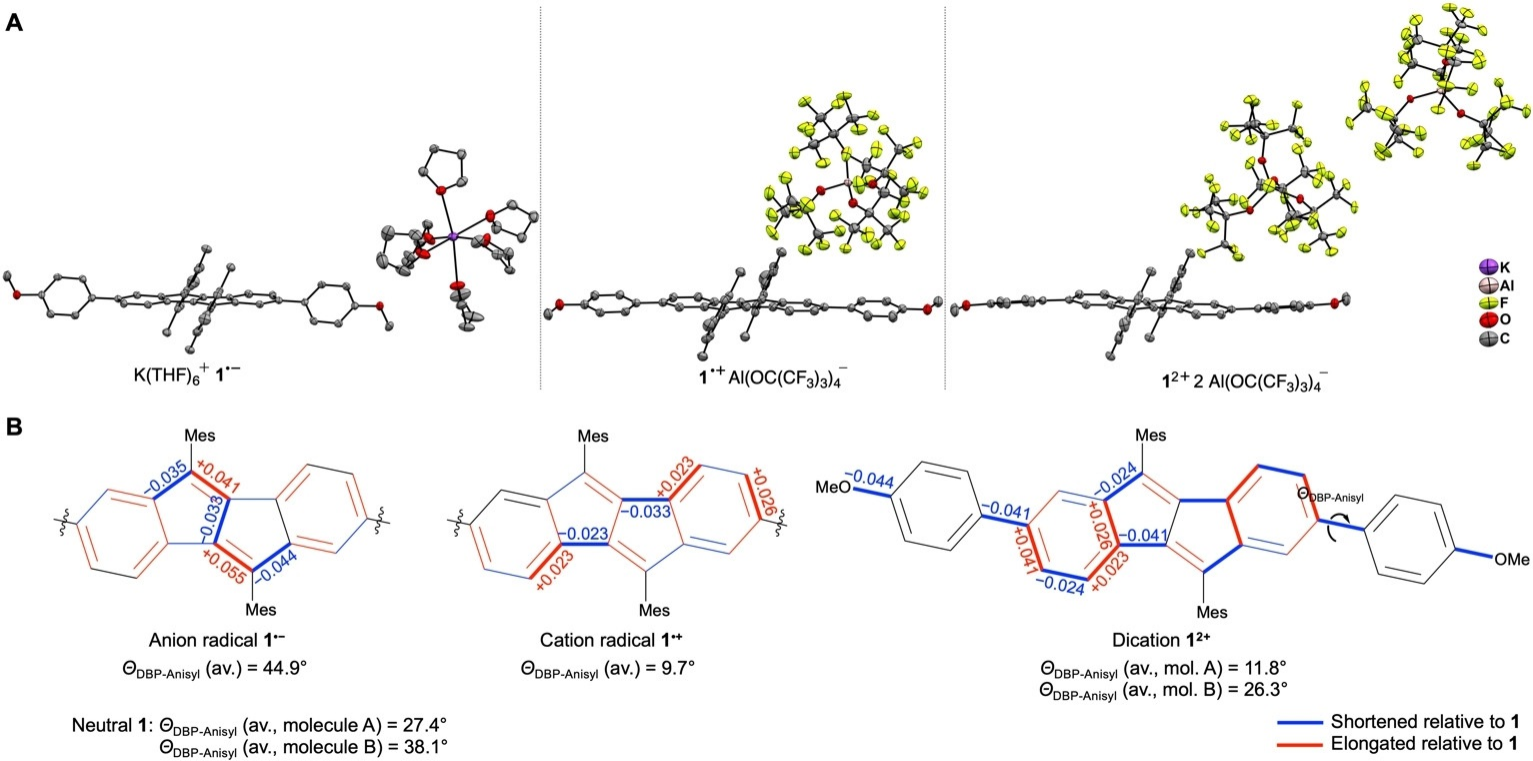
A) Molecular structures of the salts of anion radical **1^.−^**, cation radical **1^.+^** and dication **1^2+^** in the solid state (displacement ellipsoids are shown at the 50 % probability level; hydrogen atoms are omitted for clarity); B) Changes in bond lengths (in Å) upon going from neutral **1** to anion radical **1^.−^**, cation radical **1^.+^** and dication **1^2+^**, based on structural parameters in the solid state (blue‐colored bonds are shortened, red‐colored bonds elongated) and average dihedral angles *Θ*_DBP‐Anisyl_ for the torsion between the DBP unit and the anisyl substituents.

The structures of the salts of **1^.−^**, **1^.+^** and **1^2+^** are shown in Figure 3, and selected bond lengths are listed in Table 1. To the best of our knowledge, this is only the second report of a DBP anion radical^[20]^ in the solid state and the first report on solid‐state structures of a DBP cation radical or dication. In anion radical **1^.−^**, likely due to the steric bulk of the two mesityl groups on the DBP core, the potassium ion is not coordinated by the DBP unit, but by six THF molecules instead. This stands in contrast to the only other DBP anion radical reported in the literature with two Si(*i*Pr)_3_ (TIPS) substituents on the DBP core, where the potassium ion was located above the center of the DBP framework and furthermore unsymmetrically coordinated with two THF molecules.^[20]^ Due to this the geometry of the DBP unit **1^.−^** remains planar, which provides an ideal situation to assess its increased aromaticity by comparison of the C−C bond lengths with neutral **1**. A similar situation was encountered for both the cation radical **1^.+^** as well as the dication **1^2+^**, where the here non‐coordinating counter anions Al(OR^F^)_4_]^−^ are far removed from the DBP cores, leaving the latter planar.

**Table 1:** Bond numbering and selected bond lengths (in Å) from X‐ray crystallographic data of **1** ^[33]^ and its reduced and oxidized species **1^.−^**, **1^.+^** and **1^2+^**.

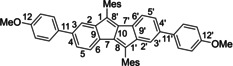				
Bond #	Anion radical **1^.−^**	Neutral **1** ^[a, 33]^	Cation radical **1^.+^**	Dication **1^2+^** ^[a]^
1	1.455(5)	1.490	1.471(3)	1.466
2	1.398(4)	1.380	1.381(3)	1.372
3	1.392(4)	1.411	1.412(3)	1.417
4	1.417(4)	1.397	1.416(3)	1.438
5	1.396(5)	1.394	1.380(3)	1.370
6	1.390(5)	1.378	1.401(3)	1.401
7	1.446(4)	1.461	1.438(3)	1.420
8	1.412(4)	1.357	1.374(3)	1.376
9	1.437(4)	1.424	1.435(3)	1.450
10	1.434(4)	1.467	1.472(3)	1.469
11	1.484(4)	1.483	1.475(3)	1.442
12	1.374(4)	1.374	1.361(2)	1.330
1’	1.446(5)	–^[b]^	1.474(3)	–^[b]^
2’	1.400(4)	–^[b]^	1.375(3)	–^[b]^
3’	1.404(4)	–^[b]^	1.414(3)	–^[b]^
4’	1.402(4)	–^[b]^	1.423(3)	–^[b]^
5’	1.390(5)	–^[b]^	1.379(3)	–^[b]^
6’	1.395(5)	–^[b]^	1.401(3)	–^[b]^
7’	1.446(4)	–^[b]^	1.428(3)	–^[b]^
8’	1.398(4)	–^[b]^	1.374(3)	–^[b]^
9’	1.435(4)	–^[b]^	1.436(3)	–^[b]^
11’	1.485(4)	–^[b]^	1.460(3)	–^[b]^
12’	1.379(4)	–^[b]^	1.352(2)	–^[b]^

[a] Two independent molecules A and B in the asymmetric unit, given is the arithmetic mean. The bond lengths errors for A and B amount to (2) or (3), resulting in an average error of (2). [b] Symmetry‐equivalent.

Most instructive regarding the (anti)aromaticity of the DBP core is a comparison of the C−C bond lengths in neutral **1**, anion radical **1^.^** ^<**M‐>**^ and oxidized species **1^.+^** and **1^2+^**. In neutral **1**, the small bond length alternation with C−C bond length between 1.378 and 1.424 Å in the six‐membered rings of the DBP units (bonds 2–6 and 9, Table 1) clearly indicate their aromatic character. In the five‐membered rings, the double bond (bond 8: 1.357 Å) and single bonds (bonds 1: 1.490 Å, 7: 1.461 Å and 10: 1.467 Å) are more localized due to the slight antiaromatic character of the pentalene core. Upon both reduction or oxidation, the C−C bond lengths in the five‐membered rings become more equalized in **1^.−^**, **1^.+^** and **1^2+^**. This is shown in detail by a red (elongated) or blue (shortened) coloring of the respective bonds in Figure 3 B including the absolute change in bond lengths compared to **1** for all bonds experiencing a change of more than 0.02 Å. In the bis‐TIPS‐substituted DBP anion radical reported in the literature,^[20]^ the same tendency of bond lengths equilibration in the pentalene core was observed compared to the neutral species.

Furthermore, the dihedral angles *Θ*_DBP‐Anisyl_ between the DBP unit and the anisyl substituents significantly change upon reduction or oxidation (Figure 3 B). In anion radical **1^.−^** with a value of 44.9°, this angle is larger than in neutral **1** with 27.4° for molecule A and 38.1° for molecule B. This indicates a reduced resonance with the anisyl group in the anion radical state, which can be rationalized with the electron‐rich nature of the anisyl substituent. The opposite is the case in cation radical **1^.+^** and dication **1^2+^**. Here, the dihedral angles are reduced to 9.7° for **1^.+^** and 11.8° respective 26.3° for molecule A and B of **1^2+^**, respectively. This shows that the positive charge(s) are partially delocalized over the anisyl groups. This is also manifested in shortened lengths of bonds 11 and 12 (and 11’ and 12’) in **1^.+^** and **1^2+^** compared to **1**. In particular, in dicationic **1^2+^**, these bonds are shortened by 0.041 and 0.044 Å, respectively, assuming partial double bond character.

Spectroelectrochemical measurements on DBP derivative **1** (see Supporting Information) showed an increase in the longer wavelength absorption maxima and absorption onset upon oxidation, indicative of a reduced band gap, while the intensity of the main absorption band centered around 340 nm decreased. Upon reduction, the intensities of all absorption bands increased, in particular the short‐wavelength bands below 300 nm.

To further assess the (anti)aromatic character of the DBP units we calculated NICS[^45^, ^46^] (nucleus independent chemical shift) values using B3LYP/6‐31G* (Table 2). For neutral **1**, the NICS(1)_iso_ value was positive above the center of the 5‐membered rings and negative for the 6‐membered rings, indicating aromatic character for the latter and antiaromatic character for the former. With all redox events, the aromaticity of the overall molecule increased. With reduction to anion radical **1^.−^**, the 5‐membered rings obtained slight aromatic character, while the aromaticity in the 6‐membered rings further increased. Strongly aromatic character was found for dianion **1^2−^**, in particular for the 5‐membered rings in the pentalene core. A similar observation can be made for the oxidation. In cation radical **1^.+^**, the 5‐membered rings have lost their antiaromatic character, and in dication **1^2+^** they even assumed a certain degree of aromaticity, while the 6‐membered rings became a bit more aromatic.

**Table 2:** NICS(1)_iso_ values for **1** and its reduced and oxidized species **1^.−^**, **1^.+^** and **1^2+^**.^[a]^

	5‐membered ring	6‐membered ring
dianion **1^2−^**	−9.05	−8.95
anion radical **1^.−^**	−2.70	−7.40
neutral **1**	5.60	−6.05
cation radical **1^.+^**	0.40	−6.00
dication **1^2+^**	−3.35	−6.65

[a] B3LYP/6‐31G* on PBEh‐3c (**1^.+^** and **1^2+^**)‐ or B3LYP/6‐31G** (**1**, **1^.^** ^−^ and **1^2^** ^−^)‐optimized geometries.

The higher aromatic character upon reduction to **1^.−^** and particularly **1^2−^** compared to oxidized **1^.+^** and **1^2+^** can be explained by the lack of conjugation to the anisyl substituents in the anionic forms. Thereby, the additional electron(s) are mostly localized on the DBP core and lead to a strong increase in aromaticity. In cations **1^.+^** and **1^2+^**, on the other hand, conjugation to the anisyl substituents is significant, as discussed above in the context of the geometrical parameters of the solid‐state structures, and the positive charges are only partially localized on the DBP core and less strongly increase its aromaticity.

**Figure 4:**
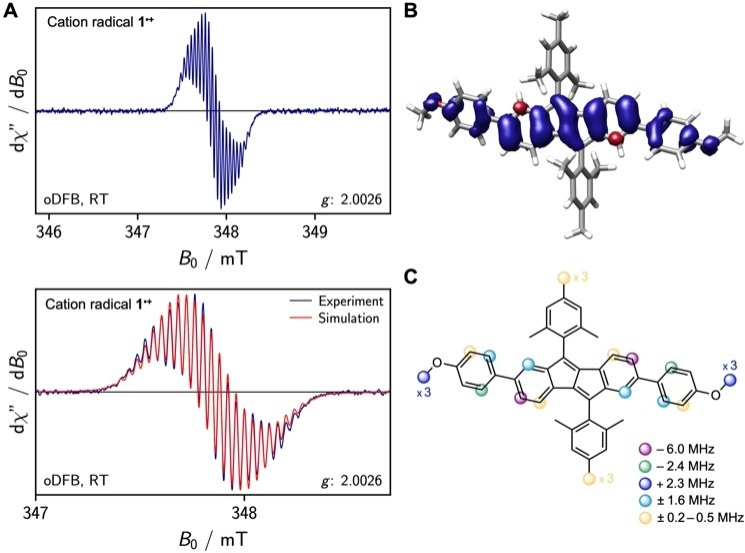
A) Continuous‐wave X‐band EPR spectrum of cation radical **1^.+^** in *o*‐DFB acquired at room temperature with a modulation amplitude of 0.005 mT (top) and best numerical simulation of the data (bottom); B) Visualization of the spin density predicted by DFT calculations; C) Assignment of the calculated hyperfine coupling constants to the protons of the structure (only hyperfine couplings with |*a*_iso_|≥0.3 MHz are shown. For hyperfine coupling constants predicted to have a positive sign, the round marker is shown above the molecular skeleton).

Electron paramagnetic resonance (EPR) spectra provided insight into the location of the spin density in cation radical **1^.+^**. The continuous wave spectrum obtained for **1^.+^** in *o*‐DFB at room temperature is shown in Figure 4 A. The spectrum is characterized by a pronounced hyperfine pattern, resulting from coupling of the unpaired electron spin to the protons of the structure. Consistent with previous literature results,^[13]^ a *g*‐value of 2.0026 was determined by numerical simulation of the data using EasySpin^[47]^ functions in combination with a home‐written MATLAB fitting routine. As shown in Figure 4 B, the spin density (predicted from DFT calculations) is distributed mainly over the DBP core, but also the anisyl substituents. This is in line with the interpretation of the solid‐state structure of **1^.+^**, which showed the anisyl groups to participate in the charge and spin delocalization.

The choice of the proton couplings accounted for in the simulations was guided by the results from DFT calculations (UKS B3LYP/EPRII) of the EPR parameters using ORCA. The protons with calculated hyperfine coupling constants larger than |*a*_iso_|≥0.3 MHz are indicated in Figure 4 C. The computed values were used as starting parameters for the numerical fit, where the ten protons with hyperfine coupling constants between ±(0.2–0.5) MHz were assumed to contribute only to the linewidth and were not explicitly accounted for. The signs of the hyperfine coupling constants were taken from DFT, since the simulation is only sensitive to the absolute values. The best fit to the experimental data, as shown in Figure 4 A (bottom), was obtained for the following hyperfine coupling constants: −4.5 MHz (×2), −2.4 MHz (× 2), +2.1 MHz (× 6), and +1.1 MHz (×4).

### Reduction of bent DBP‐phane 2

**Figure 5:**
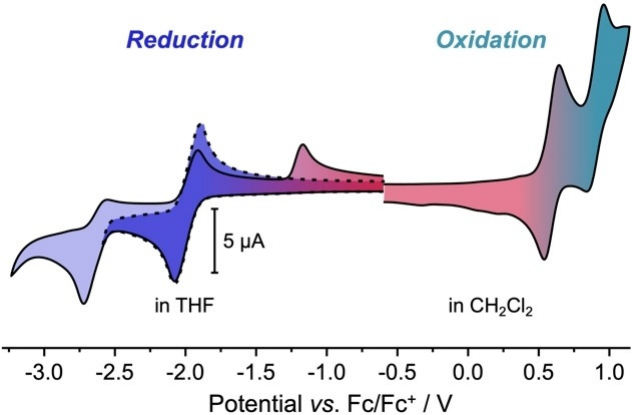
Cyclic voltammograms of DBP‐phane **2** in THF (reduction) and CH_2_Cl_2_ (oxidation) (1 mm, 0.1 m *n*Bu_4_NPF_6_, scan rate 0.1 V s^−1^, glassy carbon electrode, potentials given versus Fc/Fc^+^ as internal standard).

Finally, we were interested to find out what effect bending of the DBP unit played in its chemical reduction or oxidation. DBP‐phane **2** (for structure see Figure 1 C) was synthesized as previously described.^[33]^ Compared to planar **1**, the oxidation potentials of **2** are only slightly shifted to lower half‐wave potentials of *E*_1/2_=0.59 and 0.90 V versus Fc/Fc^+^ (in CH_2_Cl_2_), with the first oxidation being reversible and the second quasi‐reversible (Figure 5). Applying similar oxidation conditions as shown in Scheme 1 to DBP‐phane **2**, however, did not provide single crystals suitable for X‐ray diffraction, but decomposition seemed to occur, even at low temperatures.

The reductions are more strongly influenced by the bending of the DBP. The first reduction was reversible, when the potential was reversed before initiating the second reduction process. With *E*_1/2_=−1.99 V vs. Fc/Fc^+^ its half‐wave potential was shifted to higher potentials by 0.14 V compared to planar **1**. The second reduction was irreversible with a cathodic peak potential of *E*_cp_=−2.72 V vs. Fc/Fc^+^ (compared to −2.77 V for **1**), which can be seen from the additional peak in anodic scan direction at −1.17 V.

**Figure 6:**
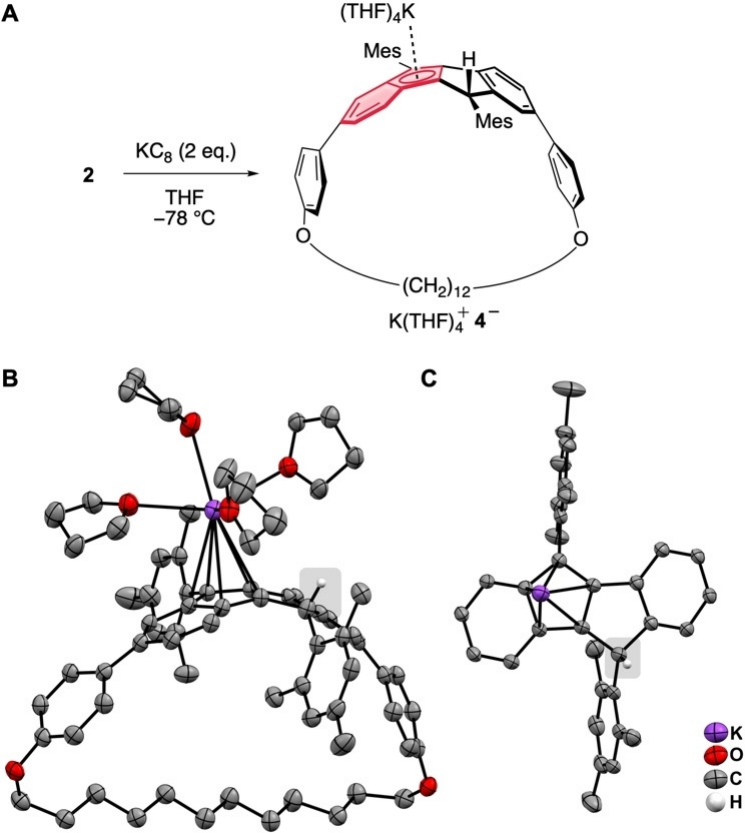
A) Reduction of DBP‐phane **2**; B) Molecular structure of the reduction product K(THF)_4_^+^ **4^−^** in the solid state (displacement ellipsoids are shown at the 50 % probability level; hydrogen atoms are omitted for clarity); C) Close‐up view of the reduced DBP core showing the tetrahedral C‐atom at the right‐hand five‐membered ring (highlighted by a grey box) and the *η* ^5^‐coordination of the K‐atom.

In order to shed light on the second irreversible reduction process in DBP‐phane **2**, we reduced this compound with two equivalents of KC_8_ in THF (Figure 6 A). After filtration, red crystals were obtained from a concentrated THF solution layered with pentane stored at −40 °C.

Interestingly, the reduced product was neither the DBP‐phane anion radical nor its dianion. Instead, single‐crystal XRD revealed the structure shown in Figure 6 B. Apparently, the dianion **2^2−^** had abstracted a proton from—most likely—a THF solvent molecule to form anion **4^−^**, where one of the 5‐membered rings was reduced to a cyclopentadiene with an sp^3^‐hybridized carbon atom. The deprotonation of THF by strong bases is a known process.^[48]^ This was evidenced by the molecular structure of the reduction product K(THF)_4_^+^ **4^−^** in the solid state (Figure 6 B). In the crystal structure, the potassium ion is *η* ^5^‐coordinated by the cyclopentadienide moiety with an additional four complexed THF molecules. The other five‐membered ring contains the tetrahedral carbon atom, as can be seen from the close‐up view in Figure 6 C. This proton abstraction is reminiscent of the reported reaction of a dianionic DBP species with Cr(CO)_3_(CH_3_CN)_3_, where proton abstraction from a CH_3_CN ligand led to a compound similar to **4^−^** with Cr(CO)_3_ *η* ^5^‐coordinated to the reduced five‐membered ring and a tetrahedral C‐atom in the other five‐membered ring.^[19]^ The reason for the instability of a dianion **2^2−^** may lie in the fact that *η* ^5^‐coordination of two potassium ions is not possible due to the incorporation of the DBP into a cyclophane structure. Coordination of the first potassium ion is hindered by the two mesityl groups (see structure of [K(THF)_6_]^+^ **1^.−^** above), and the second potassium ion would need to bind from the bottom side of the DBP, which is sterically inaccessible. In all reported solid‐state structures of DBP dianions in the literature with alkaline counter cations, both metals coordinate from opposite sides of the DBP.[^16^, ^18^, ^19^] Furthermore, due to the strained structure of DBP‐phane **2**, a planarization of the DBP unit upon twofold reduction is not possible, which would be required to fully profit from the obtained aromatic character. The fact that the potassium ion does coordinate to one five‐membered ring in K(THF)_4_^+^ **4^−^** stands in contrast to the solid‐state structure of the planar anion radical in the salt [K(THF)_6_]^+^ **1^.−^** (see above). It is likely due to one of the mesityl groups being bent away from the five‐membered rings in **4^−^** because of the tetrahedral geometry of that carbon atom, providing sufficient space for the K(THF)_4_^+^ moiety to bind.

## Conclusions

We herein investigated the chemical oxidation and reduction of bis‐anisyl‐substituted DBP **1** and DBP‐phane **2**. Their molecular structures in the solid state are the first examples for a DBP cation radical and dication, and the second example of a DBP anion radical. CC‐bond length analyses and NICS calculations showed that the antiaromaticity of **1** decreased upon both reduction and oxidation, in particular in the five‐membered rings, rendering the charged species aromatic. In the oxidized forms, the anisyl substituents participated in the charge delocalization, as seen from their bond lengths and co‐planarization. In addition, the hyperfine couplings determined by EPR spectroscopy signaled the delocalization of the spin density over the entire molecular ion and even to the anisyl moieties. For DBP‐phane **2**, the two‐fold reduction product underwent proton abstraction from a solvent molecule. This was likely due to its bent structure, which could not well accommodate the added charge, as well as the steric bulk of two mesityl substituents, hindering the coordination of two potassium cations. This study testifies to the ambipolar electrochemical character of the DBP and provides structural insight into its charged species, relevant for OFET applications.

## Experimental Section

DBP **1** and DBP‐phane **2** were synthesized as previously described.^[33]^ Details on materials and methods can be found in the Supporting Information.

**Synthesis of 1^.+^ [Al(OR^F^)_4_]^−^**: A solution of **3** [Al(OR^F^)_4_] (0.070 g, 0.043 mmol) in *o*‐DFB (3 mL) was slowly added to a solution of **1** (0.030 g, 0.047 mmol, 1.1 equiv.) in *o*‐DFB (2 mL). Upon addition the immediate formation of a dark red color was observed. The reaction mixture was stirred for a further 30 min at ambient temperature and subsequently layered with *n*‐pentane to yield dark red/black crystals suitable for scXRD analysis (0.047 g, 0.029 mmol, 67 % crystalline yield).

**Synthesis of 1^2+^ ([Al(OR^F^)_4_]^−^)_2_**: **3** [Al(OR^F^)_4_] (0.070 g, 0.043 mmol) in *o*‐DFB (3 mL) and **1** (0.014 g, 0.022 mmol, 0.5 equiv.) were dissolved in *o*‐DFB (3 mL) with immediate formation of a dark blue color. The reaction mixture was stirred for a further 30 min at ambient temperature and subsequently layered with *n*‐pentane to yield dark blue/black crystals suitable for scXRD analysis (0.042 g, 0.016 mmol, 75 % crystalline yield).

**Synthesis of [K(THF)_6_]^+^1^.−^**: A solution of **1** (107 mg, 0.16 mmol) in 2 mL THF was slowly added to a suspension of KC_8_ (22 mg, 0.16 mmol, 1 equiv.) in 2 mL THF at −78 °C. An immediate color‐change from orange to blue was observed. The reaction mixture was stirred for 15 minutes at −78 °C. The cooling bath was removed and the reaction mixture was stirred for 3 h at rt. After filtration, the solution was concentrated and stored at −40 °C to give dark blue crystals suitable for scXRD.

**Synthesis of [K(THF)_4_]^+^ 4^−^**: A solution of **2** (82 mg, 0.10 mmol) in 2 mL THF was slowly added to a suspension of KC_8_ (30 mg, 0.22 mmol, 2.2 equiv.). An immediate color‐change from orange‐red to purple was observed. The reaction mixture was stirred for 15 minutes at −78 °C. The cooling bath was removed and the reaction mixture was stirred for 1.5 h at rt. After filtration, the reaction mixture was concentrated, layered with pentane and stored at −40 °C to give dark red crystals suitable for scXRD. The crystals were embedded in a sticky solid which may be caused by the yet unidentified proton abstraction reaction.

Deposition numbers 2045958, 2045957, 2045909, and 2045910 (**1^.+^** [Al(OR^F^)_4_]^−^, **1^2+^** ([Al(OR^F^)_4_]^−^)_2_, [K(THF)_6_]^+^ **1^.−^**, and [K(THF)_4_]^+^ **4^−^**) contain the supplementary crystallographic data for this paper. These data are provided free of charge by the joint Cambridge Crystallographic Data Centre and Fachinformationszentrum Karlsruhe Access Structures service www.ccdc.cam.ac.uk/structures.

## Conflict of interest

The authors declare no conflict of interest.

## Supporting information

As a service to our authors and readers, this journal provides supporting information supplied by the authors. Such materials are peer reviewed and may be re‐organized for online delivery, but are not copy‐edited or typeset. Technical support issues arising from supporting information (other than missing files) should be addressed to the authors.

Supplementary
