# Supplementary material for: Cations and Anions of Dibenzo[a,e]pentalene and Reduction of a Dibenzo[a,e]pentalenophane
Source: Chemistry. 2021 Feb 12;27(15):4964–70. doi: 10.1002/chem.202005131 (PMC7986162; doi:10.1002/chem.202005131)
Supplement: Supplementary file 1 — Supplementary [file CHEM-27-4964-s001.pdf]

Chemistry–A European Journal

Supporting Information

Cations and Anions of Dibenzo[*a*,*e*]pentalene and Reduction of a Dibenzo[*a*,*e*]pentalenophane

Mathias Hermann,[a] Tobias Böttcher+,[b] Marcel Schorpp+,[b] Sabine Richert,[c] Daniel Wassy,[a] Ingo Krossing,[b, d, e] and Birgit Esser*[a, d, e]

Contents

1 Synthetic Manipulations S2
2 EPR measurements S7
3 Cyclic Voltammetry........ S8
4 Spectroelectrochemical measurements........ S11
5 Single-Crystal X-ray Diffraction S15
5.1 Bond length analysis........ S19
5.2 Torsion angles S22
6 DFT Calculations........ S23
7 References........ S28

1 Synthetic Manipulations

Dibenzopentalenes **1** and **2** have been synthesized as previously described.[1]

All reactions and manipulations were carried out under an inert argon atmosphere, using standard Schlenk-line and glovebox techniques (box atmosphere kept below 1 ppm H_2O/O_2). Glassware has been stored over-night in an oven set to 180°C and flame dried under vacuum prior to use. **3** $[Al(OR^F)_4]$ was prepared according to reported procedure.[2] Pentane was collected from a solvent purification system (SPS) and oxygen removed by purging with Argon. Solvents were dried and distilled under an argon atmosphere: tetrahydrofuran (THF) over sodium wire, 1,2-Difluorobenzene (*o*-DFB) and *n*-pentane were refluxed over CaH_2. All solvents were stored over activated 3 Å molecular sieves in gas tight ampoules. NMR samples were prepared inside an inert atmosphere glovebox in NMR tubes equipped with a gas-tight J.YOUNG valve. ^{1}H, ^{13}C, ^{19}F, ^{27}Al spectra were acquired either on a Bruker Biospin Avance II+ 400 MHz WB, a Bruker Avance 200 MHz or a Bruker Avance III HD 300 MHz spectrometer. ^{1}H and ^{13}C NMR spectra are reported relative to TMS and were calibrated to residual solvent resonances.[3] Data analysis was performed using the Bruker TOPSPIN 3.5 software. The broad resonance at δ = 70 ppm observed in ^{27}Al-NMR spectra corresponds to a background from Al-nuclei in the probe head.

Synthesis of $1^{\cdot+}[Al(OR^F)_4]^-$

A solution of **3** $[Al(OR^F)_4]$ (0.070 g, 0.043 mmol) in *o*DFB (3 mL) was slowly added to a solution of **1** (0.030 g, 0.047 mmol, 1.1 eq.) in *o*-DFB (2 mL). Upon addition the immediate formation of a dark red color was observed. The reaction mixture was stirred for further 30 min at ambient temperature and subsequently layered with n-pentane to yield dark red/black crystals suitable for scXRD analysis (0.047 g, 0.029 mmol, 67% crystalline yield).

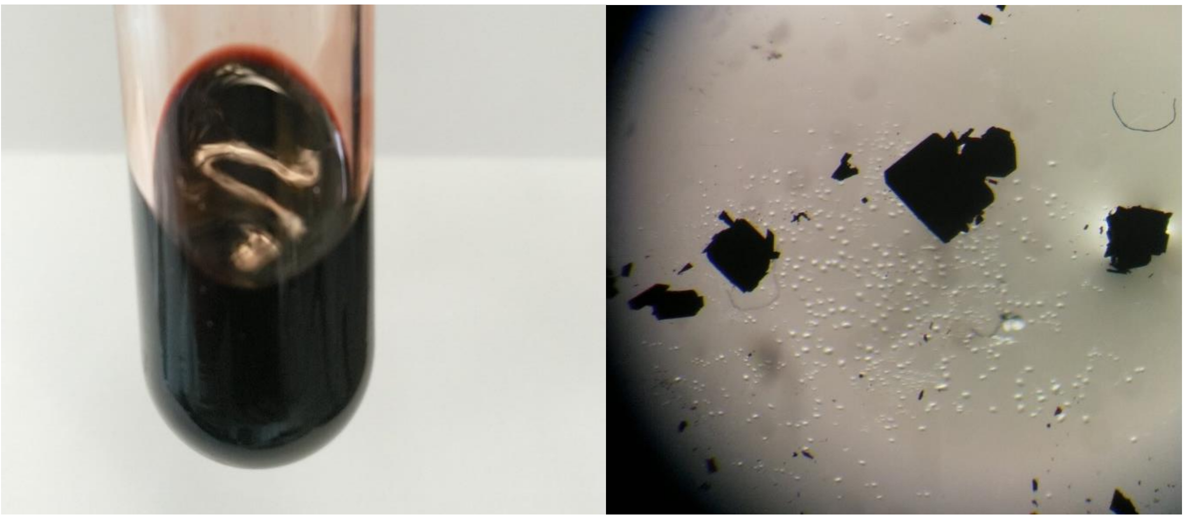

Figure S1. Images of **$1^{\cdot+}[Al(OR^F)_4]^-$** in *o*-DFB solution (left) and single crystals under the optical microscope (right).

For EPR measurements: **3** $[Al(OR^F)_4]$ (8 mg, 0.005 mmol) and **1** (16 mg, 0.024 mmol, 5 eq.) were dissolved in *o*-DFB (10 mL) to yield a 0.5 mM solution of **1**$^{\cdot+}[Al(OR^F)_4]^-$. A sufficient amount of the reaction mixture (~0.3 mL) was transferred to a Quartz-glass EPR tube equipped with a gas tight J.YOUNG valve.

Synthesis of $\mathbf{1^{2+}([Al(OR^F)_4]^-)_2}$

3 $[Al(OR^F)_4]$ (0.070 g, 0.043 mmol) in *o*DFB (3 mL) and **1** (0.014 g, 0.022 mmol, 0.5 eq.) were dissolved in *o*-DFB (3 mL) with immediate formation of a dark blue colour. The reaction mixture was stirred for further 30 min at ambient temperature and subsequently layered with n-pentane to yield dark blue/black crystals suitable for scXRD analysis (0.042 g, 0.016 mmol, 75% crystalline yield).

For NMR measurements: **3** $[Al(OR^F)_4]$ (0.070 g, 0.043 mmol) and **1** (0.013 g, 0.019 mmol, 0.45 eq.) were weighed into an NMR tube equipped with a gas tight J.YOUNG valve and dissolved in CD_2Cl_2 (0.8 mL).

^{1}H NMR (300.18 MHz, CD_2Cl_2, 298 K): δ = 7.28 (br. s, $\omega^{½}$ = 27 Hz, 6H, $C_6H_5OCH_3$), 2.51 (br. s, $\omega^{½}$ = 15 Hz 6H, Mes, *p*CH_3), 2.24 (br. s, $\omega^{½}$ = 3 Hz 12H, Mes, *o*CH_3) ppm. **^{19}F NMR** (282.45 MHz, CD_2Cl_2, 298 K) δ = −74.6 (s, 9F, $OC(CF_3)_3$), −75.7 (s, 36F, $[Al(OC(CF_3)_3)_4]^-$), −148.7 (m, AA'BB'C, 4F, phenazine^F, **3**), −154.4 (m, AA'BB'C, 2F, phenazine^F, **3**), −155.6 (m, AA'BB', 4F, phenazine^F, **3**), −161.6 (m, AA'BB', 4F, phenazine^F, **3**), −164.1 (m, AA'BB'C, 4F, phenazine^F, **3**). **^{27}Al NMR** (78.22 MHz, CD_2Cl_2, 298 K) δ = 34.5 (s, $[Al(OC(CF_3)_3)_4]^-$) ppm.

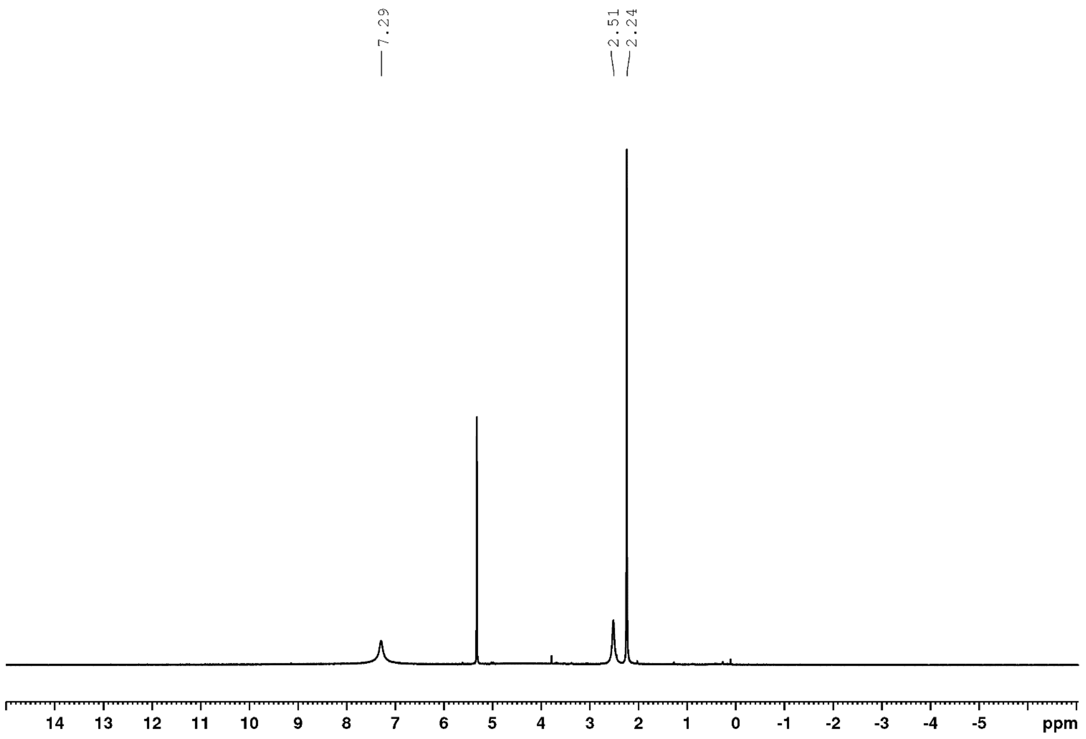

Figure S2. ^{1}H NMR spectrum of $\mathbf{1^{2+}([Al(OR^F)_4]^-)_2}$ in CD_2Cl_2 (300.18 MHz, 298 K).

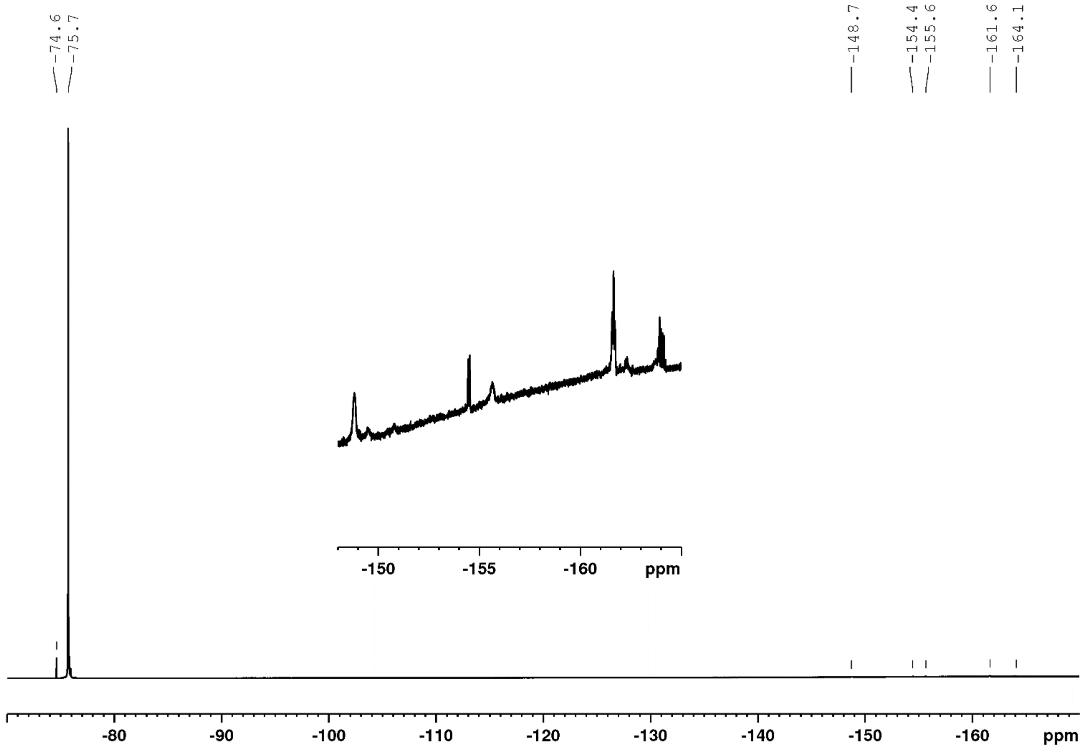

Figure S3. ^{19}F NMR spectrum of $\mathbf{1^{2+}([Al(OR^F)_4]^-)_2}$ (282.45 MHz, CD_2Cl_2, 298 K).

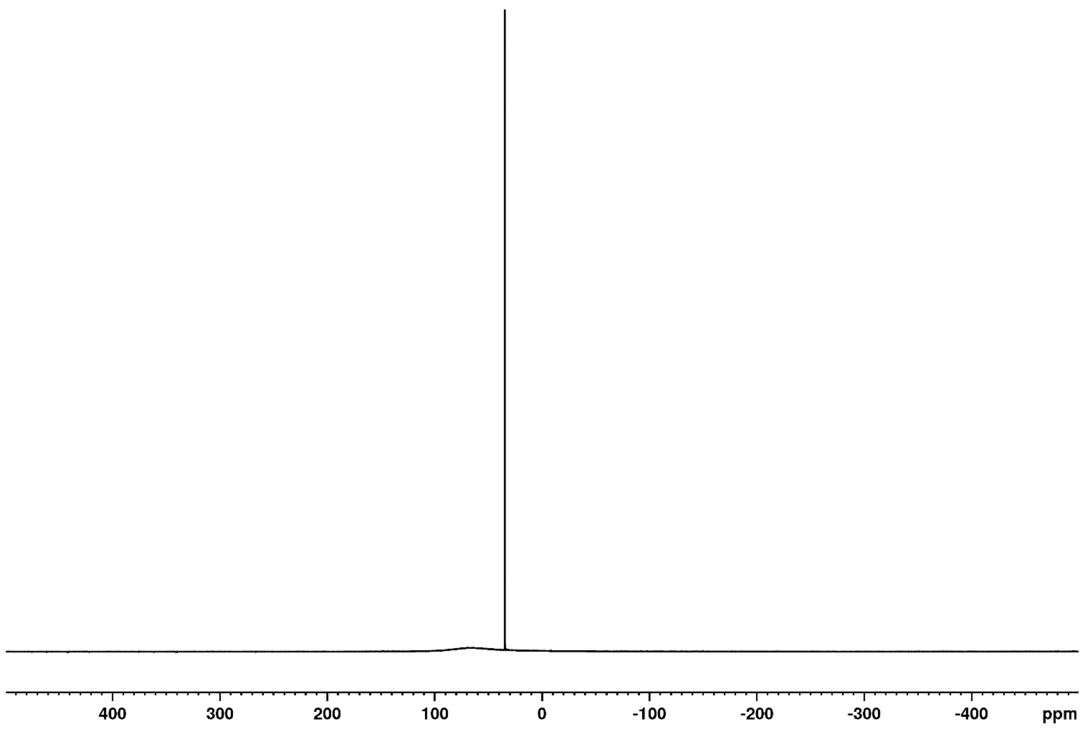

Figure S4. ^{27}Al NMR spectrum of $\mathbf{1^{2+}([Al(OR^F)_4]^-)_2}$ in CD_2Cl_2 (78.22 MHz, 298 K).

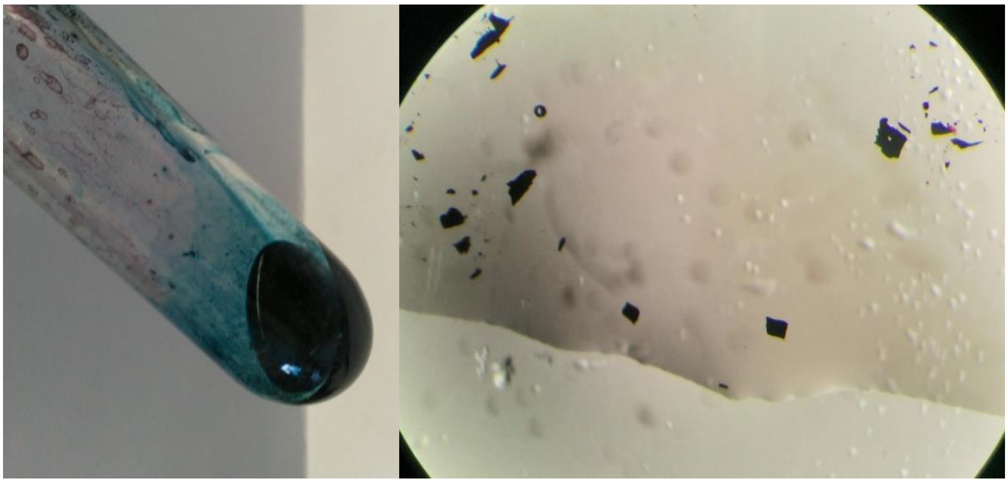

Figure S5. Images of $\mathbf{1^{2+}([Al(OR^F)_4]^-)_2}$ in *o*-DFB solution (left) and single crystals under the optical microscope (right).

Attempted Synthesis of $2^{\bullet+}[Al(OR^F)_4]^-$ and $2^{2+}([Al(OR^F)_4]^-)_2$

Syntheses were carried out in analogy to above described procedures for the single {**3** $[Al(OR^F)_4]$ (0.062 g, 0.038 mmol) in *o*-DFB (3 mL) and **2** (0.031 g, 0.040 mmol, 1.05 eq.) in *o*-DFB (2 mL)} and double {**3** $[Al(OR^F)_4]$ (0.070 g, 0.043 mmol) and **2** (0.017 g, 0.022 mmol, 0.5 eq.) in *o*-DFB (3 mL)} oxidation of **1**. The reaction mixture presumably containing mono-cationic $\mathbf{2^{\bullet+}}[Al(OR^F)_4]^-$ exhibited a dark red color while the reaction mixture presumably containing dicationic $\mathbf{2^{2+}}$ $([Al(OR^F)_4]^-)_2$ showed a dark green color. Both reaction mixtures were layered with *n*-pentane. Over the course of 12 h both reaction mixtures changed color to a dark brown with the formation of an oily residue in the bottom of the layering ampoule. Repetition of the syntheses with temperatures strictly kept below −30 °C have shown similar outcomes. The proposed reaction products of the single/double oxidation of **2** were hence deemed unstable. EPR measurements of the obtained reaction mixtures showed only broad featureless signatures which rapidly vanished over the course of the collection.

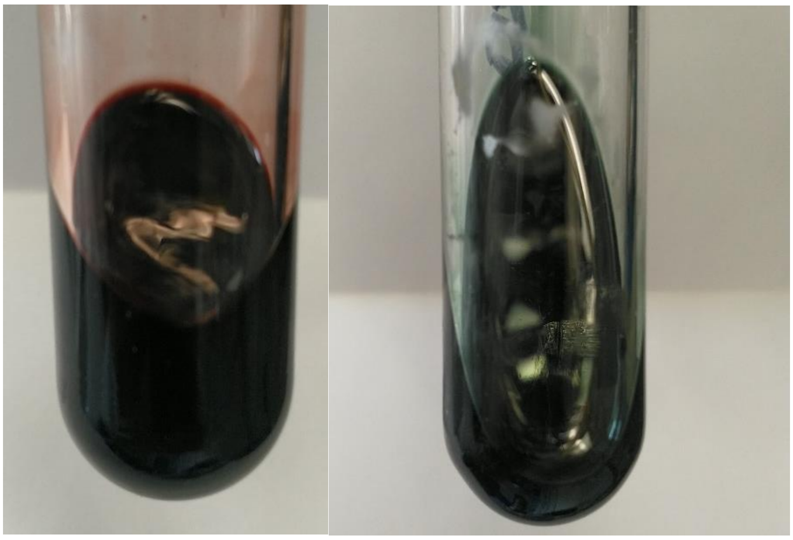

Figure S6. Images of *o*-DFB solutions presumably containing mono-cationic $\mathbf{2^{\bullet+}}[Al(OR^F)_4]^-$ (left) and dicationic $\mathbf{2^{2+}}$ $([Al(OR^F)_4]^-)_2$ (right).

Synthesis of $[K(THF)_6]^+$1$^{\bullet -}$

A solution of **1** (107 mg, 0.16 mmol) in 2 mL THF was slowly added to a suspension of KC_8 (22 mg, 0.16 mmol, 1 eq.) in 2 mL THF at −78 °C. An immediate color-change from orange to blue was observed. The reaction mixture was stirred for 15 minutes at −78 °C. The cooling bath was removed and the reaction mixture was stirred for 3 h at rt. After filtration, the solution was concentrated and stored at −40 °C to give dark blue crystals suitable for scXRD.

EPR measurement: Single crystals of $[K(THF)_6]^+$**1**$^{\bullet -}$ were collected and dissolved in THF until the color of the solution remained dark blue. The solution was transferred to a Quartz-glass EPR tube and sealed with Critoseal.

N.B. All attempts in making stock solutions with a defined concentration failed. The solutions turned out to be unstable and rapid discoloration from dark blue to yellow was already observed in the glove-box.

Synthesis of $[K(THF)_4]^+$4$^-$:

A solution of **2** (82 mg, 0.10 mmol) in 2 mL THF was slowly added to a suspension of KC_8 (30 mg, 0.22 mmol, 2.2 eq.). An immediate color-change from orange-red to purple was observed. The reaction mixture was stirred for 15 minutes at −78 °C. The cooling bath was removed and the reaction mixture was stirred for 1.5 h at rt. After filtration, the reaction mixture was concentrated, layered with pentane and stored at −40 °C to give dark red crystals suitable for scXRD. The crystals were embedded in a sticky solid which may be caused by the yet unidentified proton abstraction reaction.

2 EPR measurements

Room temperature continuous wave (cw) EPR spectra of the anions and cations of DBP were recorded at the Xband (9.75 GHz) on a Bruker EMXnano benchtop EPR spectrometer. The modulation frequency was set to 100 kHz and the modulation amplitude to 0.01 mT at a microwave power of 1 mW (20 dB). After data acquisition, the spectrum was baseline-corrected, frequency-corrected to 9.75 GHz and field-corrected using a carbon fibre standard with g = 2.002644[4]. The $g$value was then calculated from the centre of the experimental spectrum

according to $g = \frac{h \cdot \nu}{\beta_e B_0}$ and further confirmed by numerical simulation of the spectra using EasySpin[5] functions in MATLAB.

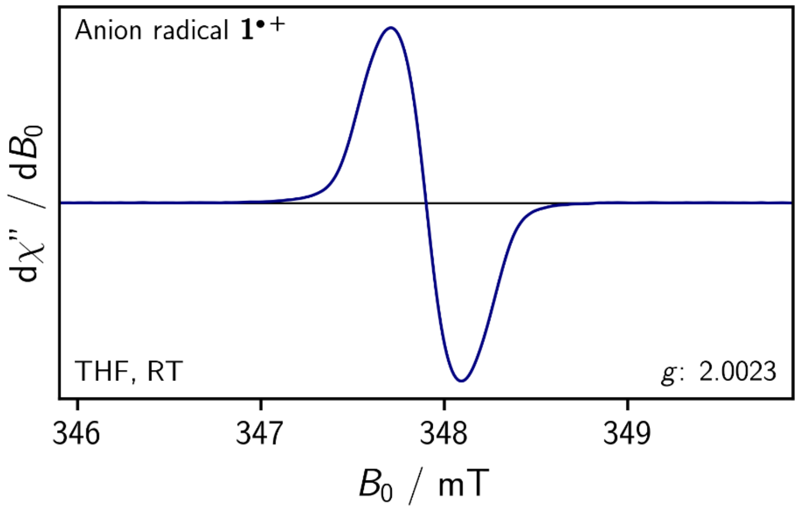

Figure S7. Continuous wave X-band EPR spectrum of **1**$^{\bullet-}$ in THF acquired at room temperature with a modulation amplitude of 0.01 mT.

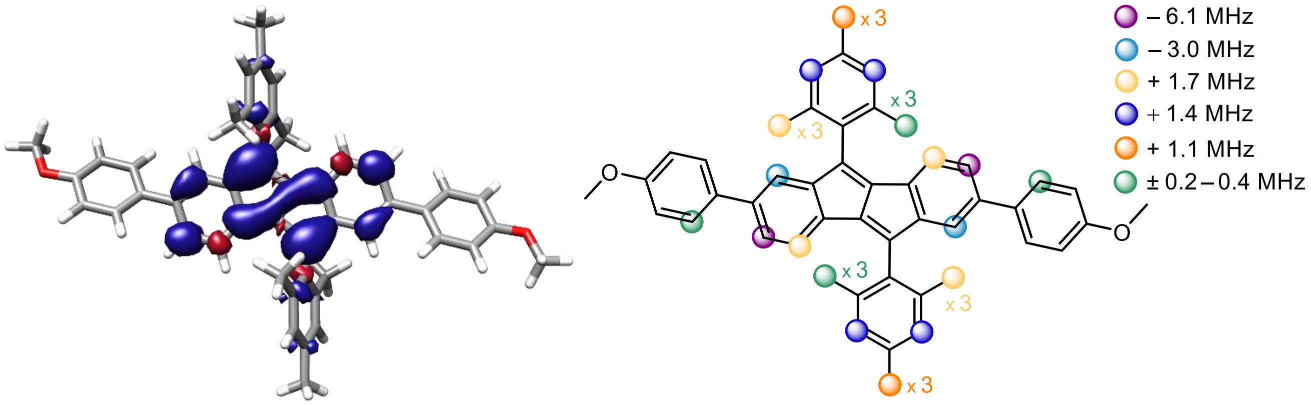

Figure 8. Visualisation of the spin density for **1**$^{\bullet-}$ predicted by DFT calculations (left) and assignment of the calculated hyperfine coupling constants to the protons of the structure (right). Only hyperfine couplings with $|a_{iso}| \geq 0.3$ MHz are shown. For hyperfine coupling constants predicted to have a positive sign, the round marker is shown above the molecular skeleton.

3 Cyclic Voltammetry

Cyclic voltammograms (CVs) were measured inside an argon filled glovebox using a PGSTAT128N by METROHM AUTOLAB. As working electrode, a glassy carbon disc electrode (2 mm diameter) was used, as counter electrode a platinum rod and as reference electrode a silver wire or an Ag/$AgNO_3$ electrode containing a silver wire immersed in an inner chamber filled with 1 M $AgNO_3$ and 0.1 M *n*-Bu_4NPF_6 in anh. CH_3CN. The analyte solution (1 mM substance concentration) contained 10 mL of solvent with 0.1 M *n*-Bu_4NPF_6 and the specified analyte concentration. The ferrocene/ferrocenium redox couple was used as internal reference. Cyclic voltammograms measured in CH_2Cl_2 were previously reported.[1]

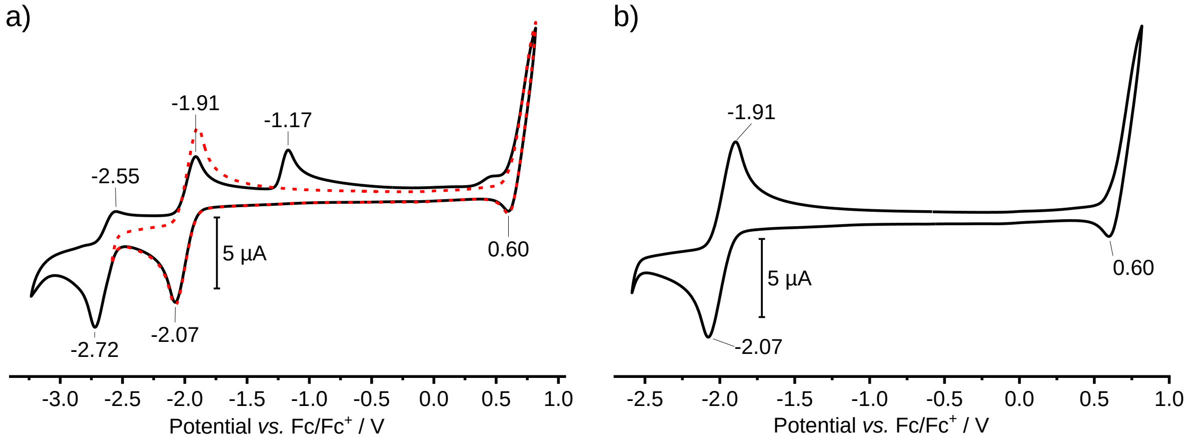

Figure S9. a) CV of **2** in THF, scan rate 0.1 V s^{-1}. The peak at −1.17 V appears because of the second reduction. Red dotted line: only the first reduction was scanned. b) Scan of the first reduction at a scan rate of 0.1 V s^{-1}, no peak in the anodic scan at −1.17 V is visible.

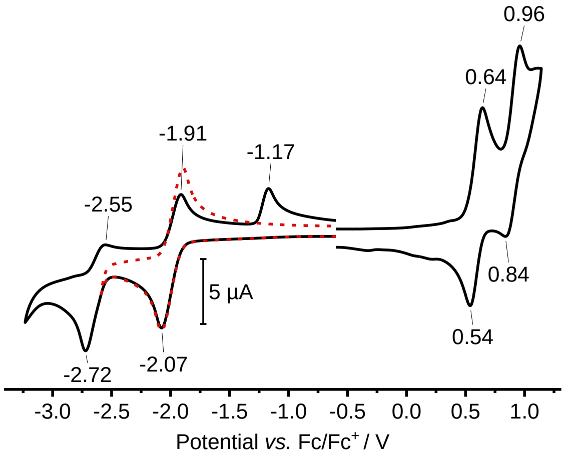

Figure S10. CV of **2** in THF (reduction) and CH_2Cl_2 (oxidation) at 0.1 V s^{-1}. Red dotted line: only the first reduction was scanned. See Figure 5 in the paper.

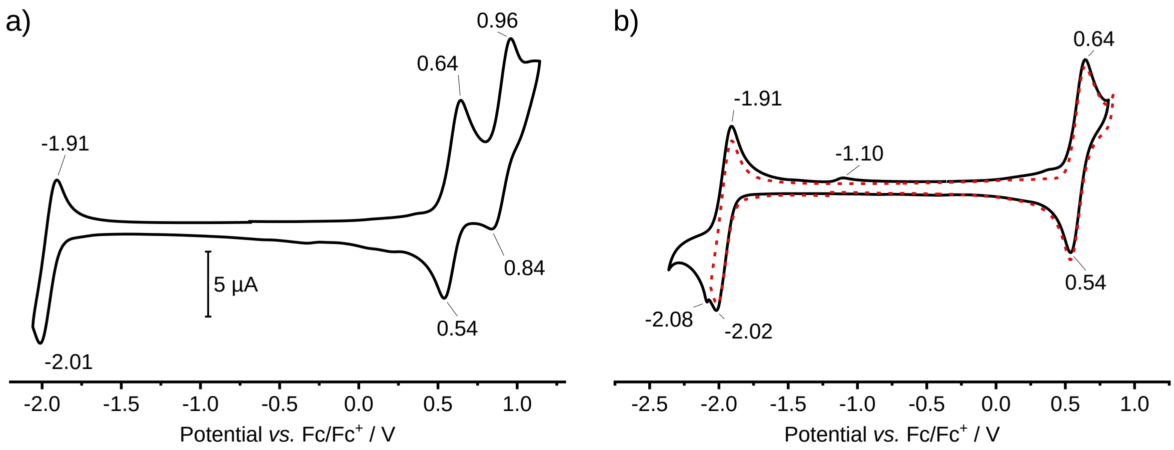

Figure S11. CVs of **2** in CH_2Cl_2 at 0.1 V s^{-1}.[1] a) Both oxidation events and only the first reduction event were scanned. b) Black line: the reduction range was extended, a second reduction event is visible at −2.08 V, with a reoxidation event at −1.10 V. Red dotted line: only the reversible range was scanned, and the reoxidation event at −1.10 V is no longer visible.

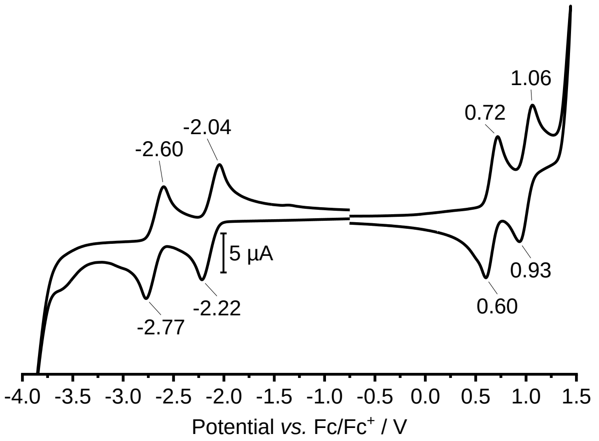

Figure S12. CV of **1** in THF (reduction) and CH_2Cl_2 (oxidation) at 0.1 V s^{-1}.

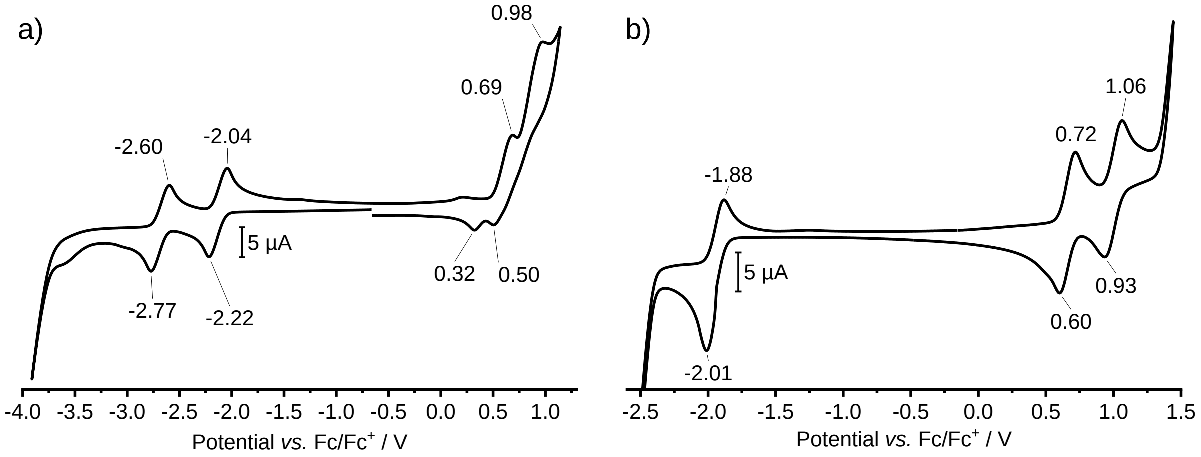

Figure S13. CV of **1** at 0.1 V s^{-1} in a) THF and b) CH_2Cl_2.

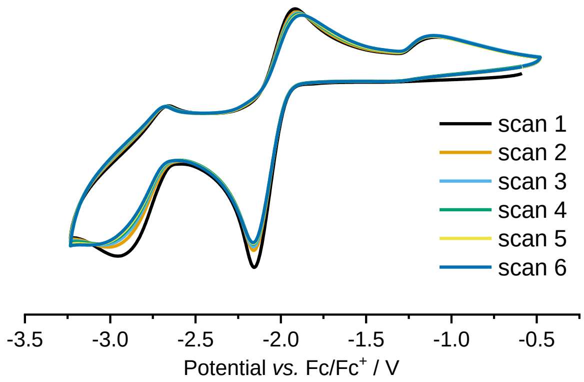

Figure S14. CV of **2** in THF in the range of −0.5 to −3.25 V *vs*. Fc/Fc^+. Six consecutive scans were performed, the new oxidation peak at −1.17 V is not reversible. It is unclear if this peak corresponds to product **4**.

4 Spectroelectrochemical measurements

Spectroelectrochemistry was performed using a combination of a PGSTAT128N potentiostat by METROHM AUTOLAB and a SEC2000 UV/VIS spectrometer by ALS Co, Ltd. A UV/VIS quartz cuvette with 1 mm path length, with a three-electrode inset was used. As working electrode a platinum mesh was used, as counter electrode a platin rod and as reference electrode a Ag/$AgNO_3$ electrode containing a silver wire immersed in an inner chamber filled with 1 M $AgNO_3$ and 0.1 M *n*-Bu_4NPF_6 in CH_3CN. To apply the desired constant potential, a chronoamperometric measurement was performed using the software METHROM Nova 2. UV/Vis spectra were collected in 30 second intervals. The analyte solution contained ~1 × 10^{-4} M analyte and 0.2 M *n*-Bu_4NPF_6 in anh. CH_2Cl_2 or THF.

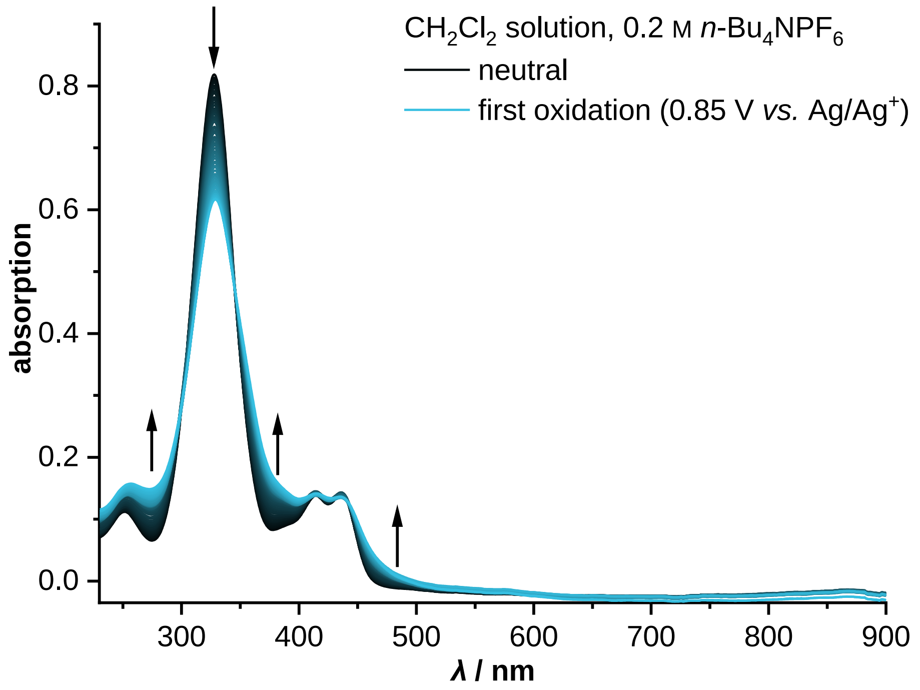

Figure S15. Spectroelectrochemical measurement of **1** in CH_2Cl_2 (1.0 × 10^{-4} M). The potential was held at 0.85 V *vs.* Ag/Ag^+ for 30 min during collection of the UV/Vis spectra.

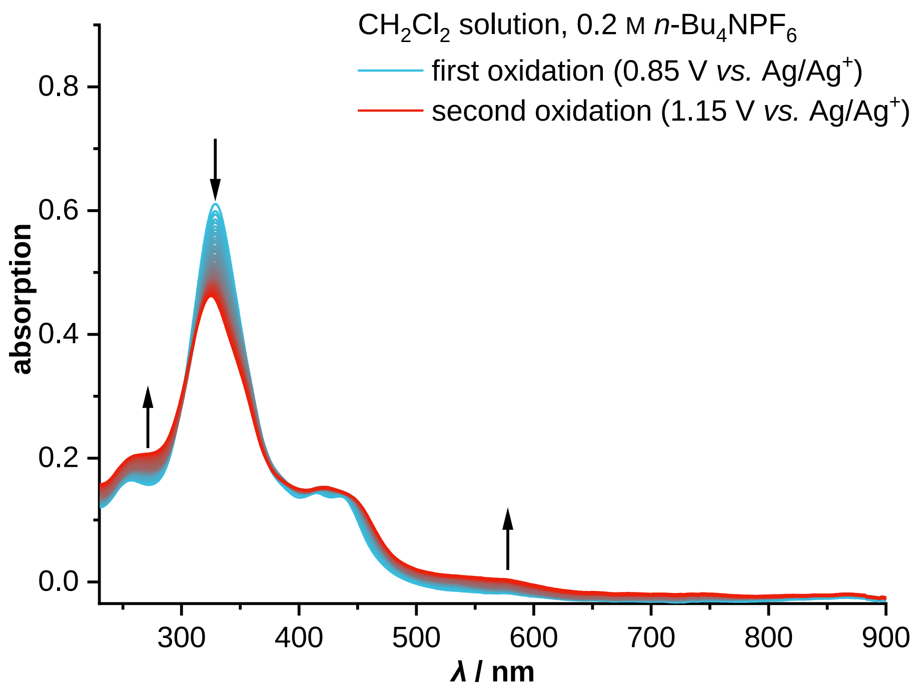

Figure S16. Spectroelectrochemical measurement of **1** in CH_2Cl_2 (1.0×10^{-4} M). The potential was held at 1.15 V vs. Ag/Ag^+ for 30 min after it was held at 0.85 V vs. Ag/Ag^+ for 30 min (Figure S15) during collection of the UV/Vis spectra.

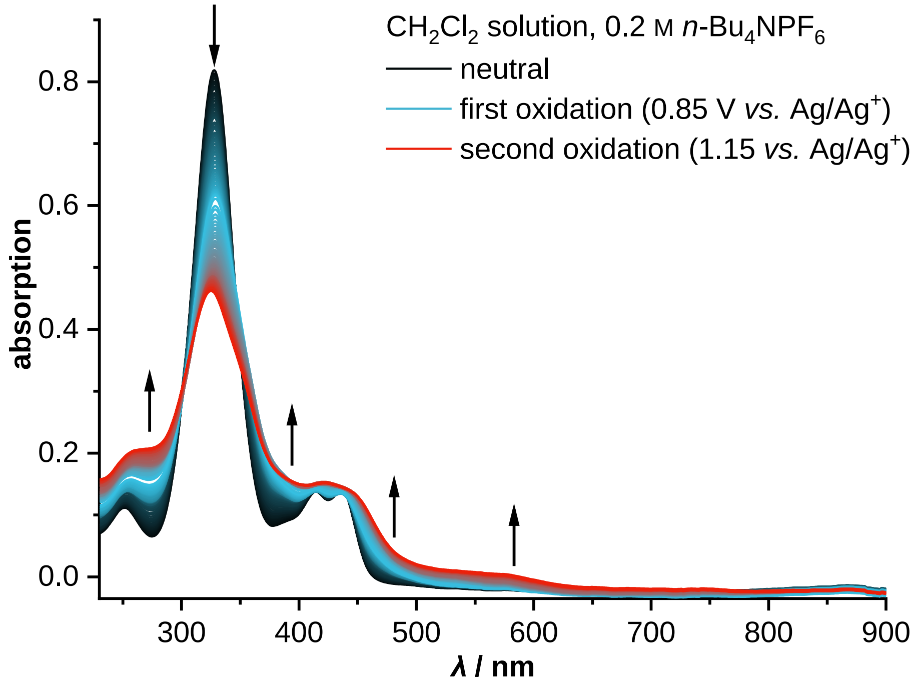

Figure S17. Spectroelectrochemical measurement of **1** in CH_2Cl_2. Combination of the data of Figure S15 and Figure S16 in one graph.

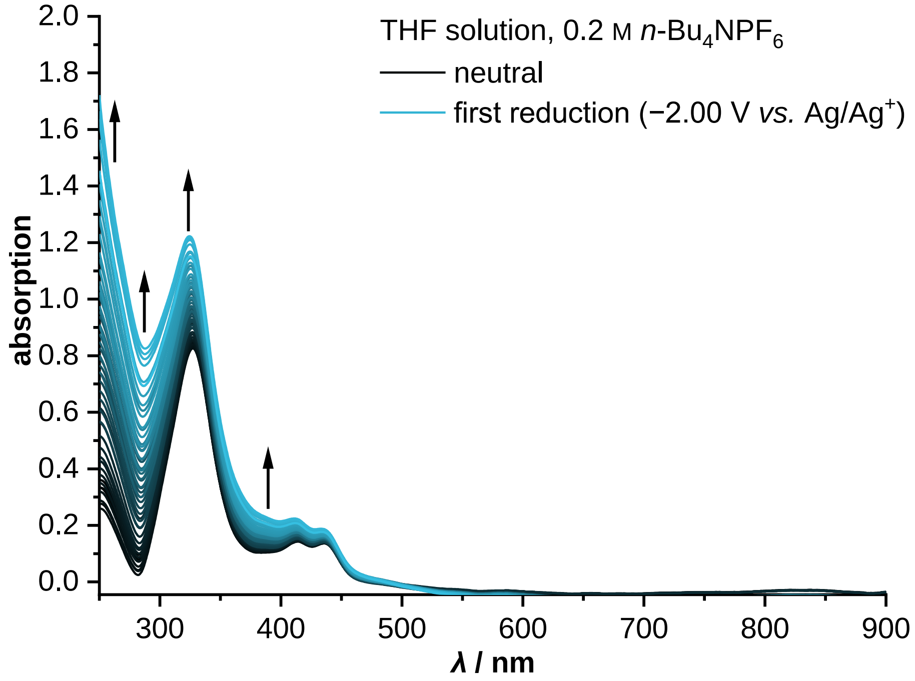

Figure S18. Spectroelectrochemical measurement of **1** in THF (9.8 × 10^{-5} M). The potential was held at −2.00 V vs. Ag/Ag^+ for 30 min during collection of the UV/Vis spectra.

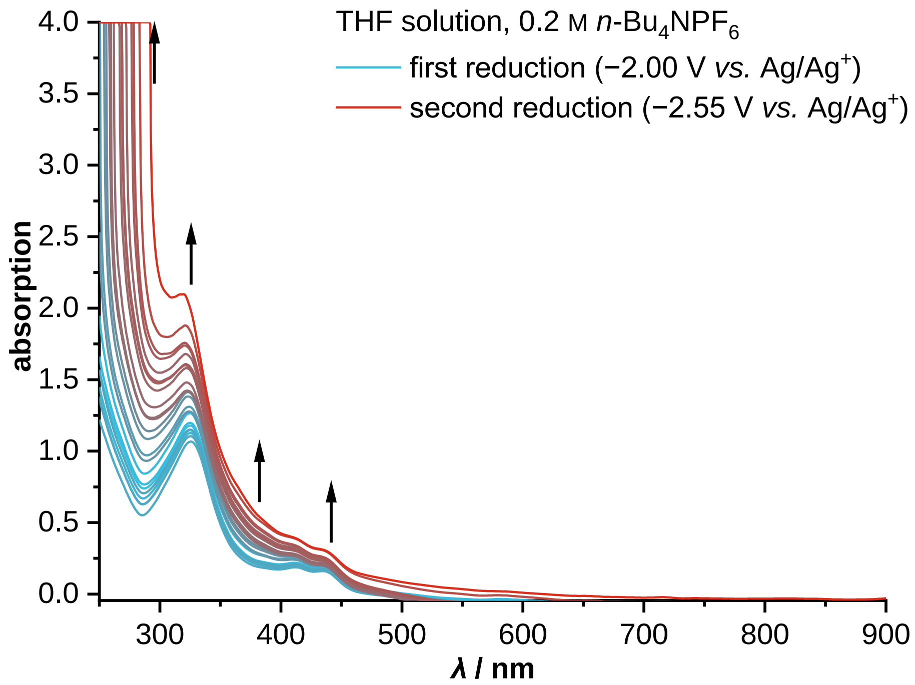

Figure S19. Spectroelectrochemical measurement of **1** in THF (9.8 × 10^{-5} M). The potential was held at −2.55 V vs. Ag/Ag^+ for 30 min after it was held at −2.00 V vs. Ag/Ag^+ for 30 min (Figure S18) during collection of the UV/Vis spectra. Decomposition occurred.

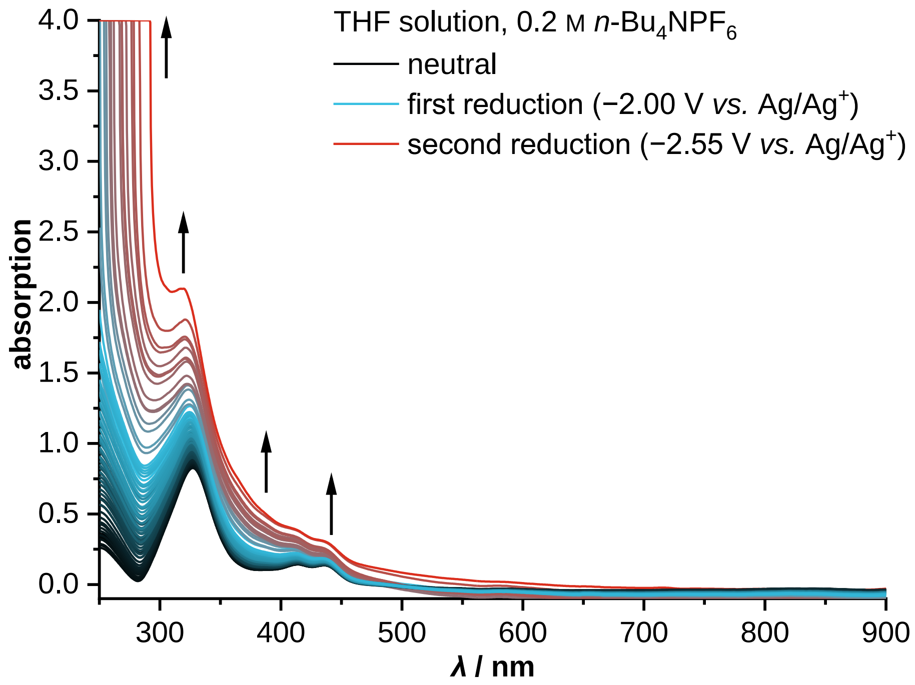

Figure S20. Spectroelectrochemical measurement of **1** in THF. Combination of the data of Figure S18 and Figure S19 in one graph.

5 Single-Crystal X-ray Diffraction

Single crystal X-ray diffraction data were collected either using a Bruker D8 VENTURE with PHOTONIII detector, fixed-Chi D8 Goniometer and INCOATEC Mo/Cu microsource or using a Bruker APEX II QUAZAR three-circle diffractometer with a microfocus sealed X-ray tube using mirror optics as monochromator and a Bruker APEX II detector. Crystals were selected under perfluoropolyether oil, mounted on 0.1 to 0.3 mm diameter CryoLoops and quench-cooled using an Oxford Cryostream 800 open flow N_2 cooling device.[6] Data were collected at 100 K using monochromated MoK_{α} (λ = 0.71073 Å) or CuK_{α} (λ = 1.54184 Å) radiation. Data processing was done with SHELXS/XL[7] and refined by least squares on weighted F_2 values for all reflections, disordering of fragments was done with the help of the implemented DSR tool.[8,9] Graphical representations have been prepared using Mercury 2020.2.0. Finalization of gathered data was done using final cif tool.[10]

The structure of neutral **1** was previously reported (CCDC 1583862).[1]

Table S1. Crystallographic data of single crystal X-ray structures.

Compound	$\mathbf{1}^{\bullet+}$[Al(OR^F)$_4$]$^-$	$\mathbf{1}^{2+}$([Al(OR^F)$_4$]$^-$)$_2$	[K(THF)$_6$]$^+$$\mathbf{1}^{\bullet-}$	[K(THF)$_4$]$^+$$\mathbf{4}^-$
CCDC number	2045958	2045957	2045909	2045910
Empirical formula	$C_{64}H_{42}AlF_{36}O_6$	$C_{80}H_{42}Al_2F_{72}O_{10}$	$C_{84}H_{113.65}KO_{11}$	$C_{74}H_{93}KO_6$
Formula weight	1617.95	2585.09	1338.49	1117.58
Temperature [K]	100(2)	100(2)	100(2)	100(2)
Crystal system	monoclinic	triclinic	triclinic	monoclinic
Space group (number)	$P2_1/c$ (14)	$P\overline{1}$ (2)	$P1$ (1)	$P2_1/c$ (14)
a [Å]	11.0964(6)	14.0362(8)	11.9702(4)	17.605(9)
b [Å]	25.6454(15)	16.1604(9)	12.1215(4)	19.746(9)
c [Å]	23.1460(12)	20.6159(12)	14.3714(5)	19.031(12)
α [Å]	90	91.753(2)	98.919(2)	90
β [Å]	91.506(2)	90.908(2)	99.769(2)	106.48(3)
γ [Å]	90	95.771(2)	112.477(2)	90
Volume [Å^3]	6584.4(6)	4649.6(5)	1842.69(11)	6344(6)
Z	4	2	1	4
ρ_{calc} [g/cm^3]	1.632	1.846	1.206	1.170
μ [mm^{-1}]	0.185	0.234	0.133	1.131
F(000)	3244	2552	725	2416
Crystal size [mm^3]	0.3×0.2×0.1	0.300×0.300×0.090	0.440×0.320×0.040	0.160×0.160×0.100
Crystal colour	black	black	blue	red
Crystal shape	plate	plate	plate	block
Radiation	MoK_α (λ =0.71073 Å)	MoK_α (λ = 0.71073 Å)	MoK_α (λ = 0.71073 Å)	CuK_α (λ = 1.54184 Å)
2Θ range [°]	3.86 to 55.81	3.95 to 52.94 (0.80 Å)	2.96 to 55.21 (0.77 Å)	5.23 to 149.39 (0.80 Å)
Index ranges	−13 ≤ h ≤ 14	−17 ≤ h ≤ 17	−15 ≤ h ≤ 15	≤ h ≤
	−33 ≤ k ≤ 33	−20 ≤ k ≤ 20	−15 ≤ k ≤ 15	≤ k ≤
	−30 ≤ l ≤ 30	−25 ≤ l ≤ 25	−18 ≤ l ≤ 18	≤ l ≤
Reflections collected	142388	293167	46588	11777
Independent reflections	15715	19050	15843	11777
	R_{int} = 0.0497	R_{int} = 0.0639	R_{int} = 0.0289	R_{int} = 0.1085
	R_{sigma} = 0.0283	R_{sigma} = 0.0256	R_{sigma} = 0.0379	R_{sigma} = 0.0616
Completeness to θ = 25.242°	99.90%	99.7%	100.0 %	96.2 %
Data / Restraints / Parameters	15715/4412/1093	19050/19061/1867	15843/597/1051	11777/782/822
Goodness-of-fit on F^2	1.053	1.101	1.022	1.053
Final *R* indexes	R_1 = 0.0442	R_1 = 0.0441	R_1 = 0.0410	R_1 = 0.0719
[I≥2σ(I)]	wR_2 = 0.1042	wR_2 = 0.0944	wR_2 = 0.0984	wR_2 = 0.1827
Final *R* indexes	R_1 = 0.0745	R_1 = 0.0550	R_1 = 0.0536	R_1 = 0.0950
[all data]	wR_2 = 0.1223	wR_2 = 0.1013	wR_2 = 0.1064	wR_2 = 0.1936
Largest peak/hole [eÅ^3]	0.35/−0.27	0.92/−0.47	0.27/−0.34	0.70/−0.39
Extinction coefficient		0.00117(15)		
Flack X parameter			0.069(12)	

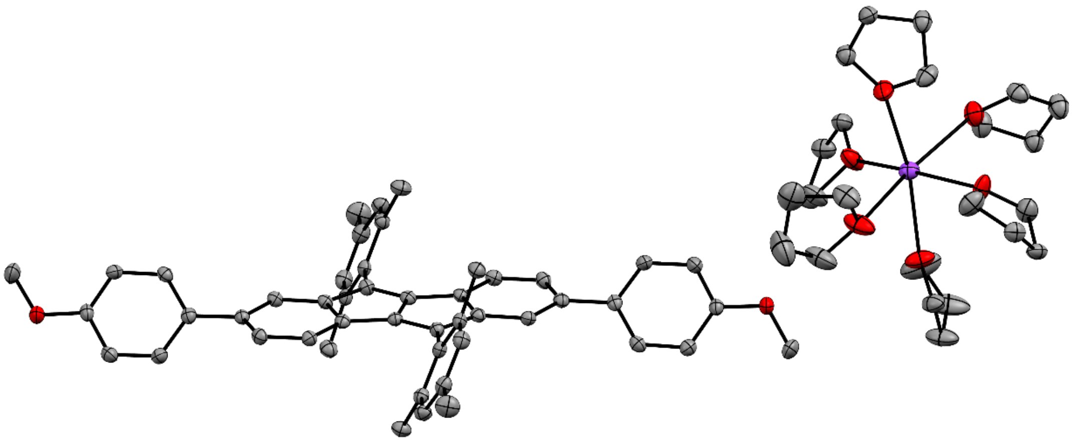

Figure S21. scXray structure of $[K(THF)_6]^+$**1**$^{\bullet-}$. Disordered THF molecules and H atoms are omitted. Thermal ellipsoids at 50% probability.

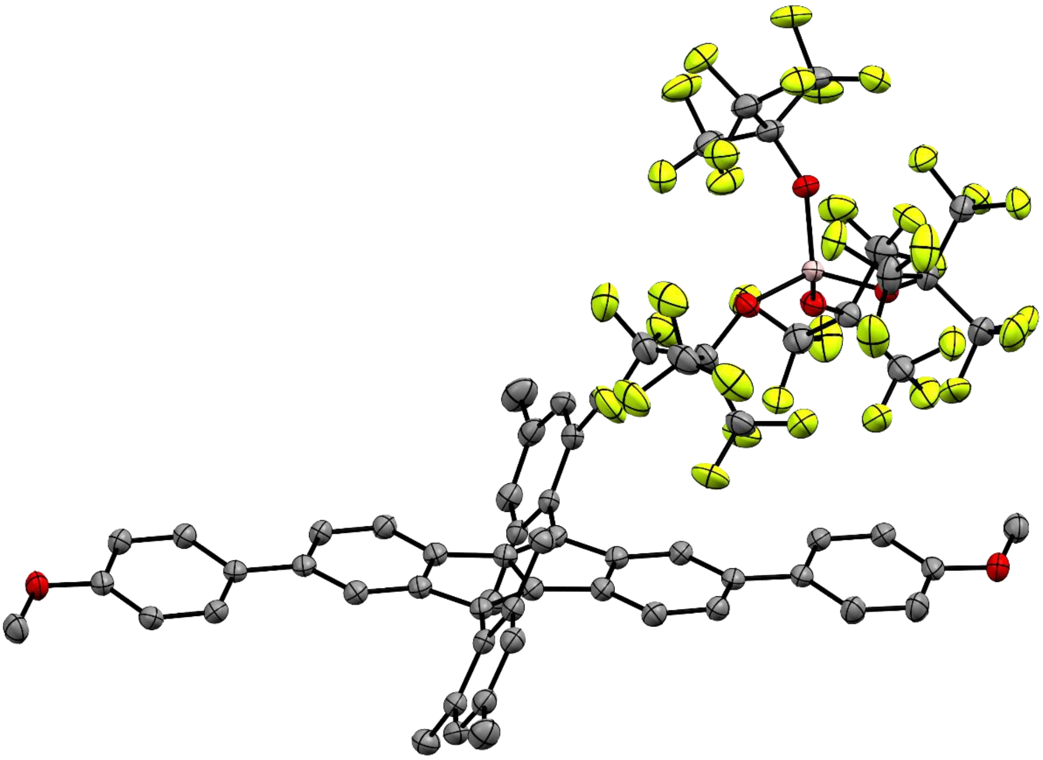

Figure S22. scXray structure of **1**$^{\bullet+}$$[Al(OR^F)_4]^-$. Disordered parts of the cation and H atoms and are omitted. Thermal ellipsoids at 50% probability.

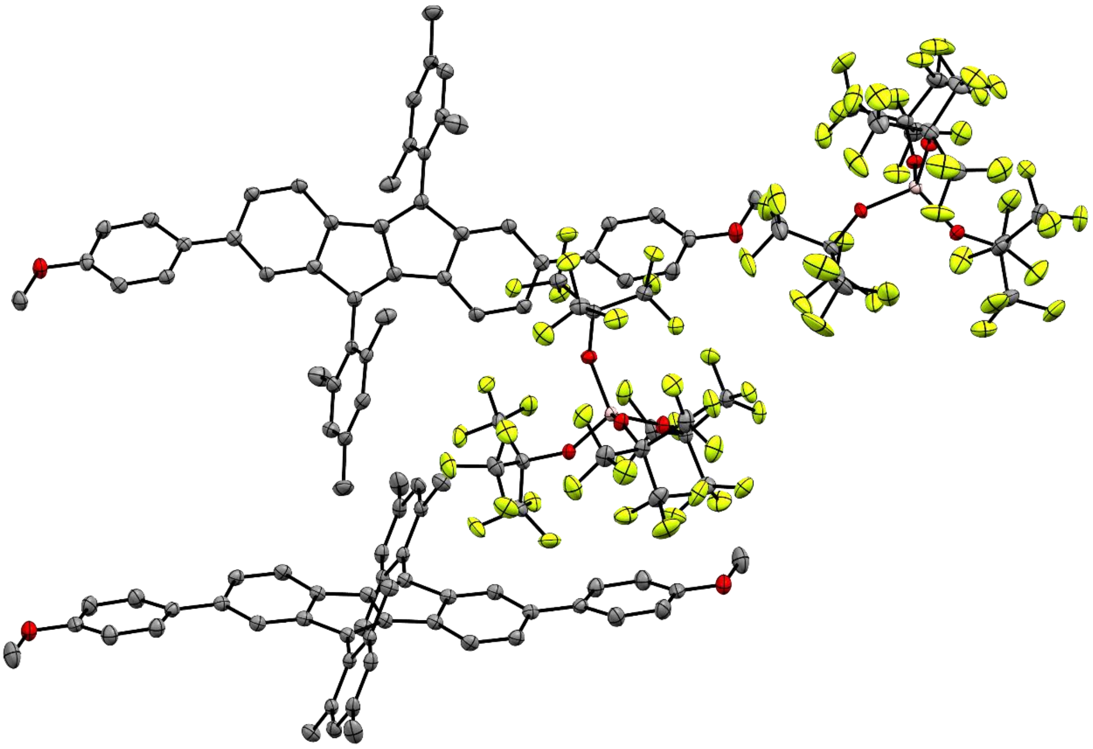

Figure S23. scXray structure of **1**$^{2+}$([Al(OR^{F})$_4$]$^{-}$)$_2$. Disordered parts of the cation and H atoms and are omitted. Thermal ellipsoids at 50% probability.

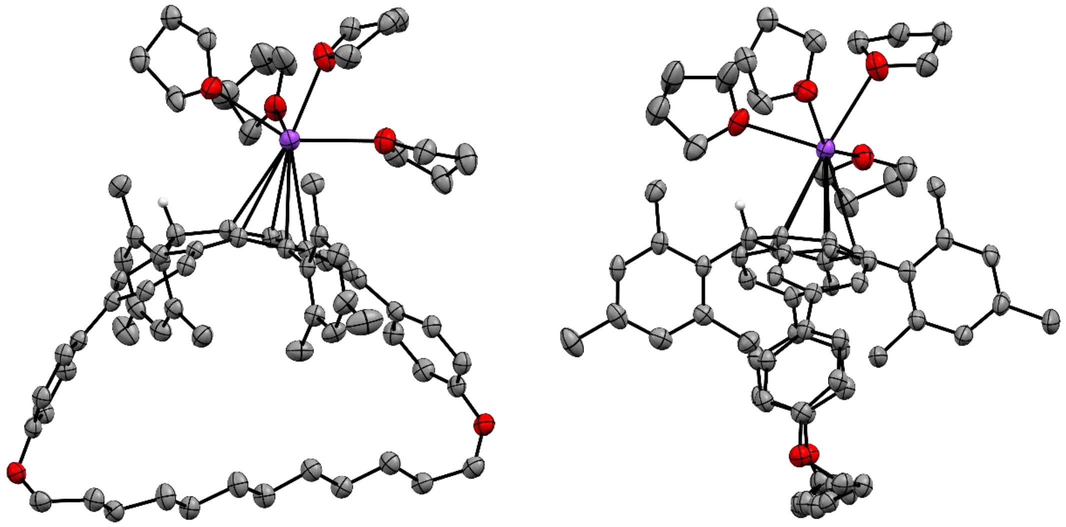

Figure S24. scXray structure of [K(THF)$_4$]$^{+}$**4**$^{-}$. Disordered THF molecules and H-atoms (except the one H-atom at the 5-memberd ring) are omitted. Thermal ellipsoids at 50% probability.

5.1 Bond length analysis

Table S2. Bond lengths of the neutral, anion radical, cation radical, and dication species of **1**. The bond lengths are given in Å, and the involved atoms with their naming according to the CIF file are stated. In case of **1** and $\mathbf{1}^{2+}$, there are two molecules in the asymmetric unit (molecule A and B), bond lengths for each molecule and the arithmetic mean are given. The bond # is assigned according to Figure S25.

	neutral (**1**)[1]					anion radical ($\mathbf{1}^{\bullet-}$)		dication ($\mathbf{1}^{2+}$)					cation radical ($\mathbf{1}^{\bullet+}$)	
#	molecule A		molecule B		average			molecule A		molecule B		average		
1	1.487(2)	C12A-C15A	1.492(3)	C12-C15	1.490(2)	1.455(5)	C17-C18	1.464(3)	C12-C15	1.468(3)	C12A-C15A	1.466(2)	1.471(3)	C36-C39
2	1.377(3)	C12A-C13A	1.382(2)	C12-C13	1.380(2)	1.398(4)	C18-C23	1.372(3)	C12-C13	1.371(3)	C12A-C13A	1.372(2)	1.381(3)	C36-C37
3	1.408(2)	C8A-C13A	1.413(3)	C8-C13	1.411(2)	1.392(4)	C22-C23	1.416(3)	C8-C13	1.417(3)	C8A-C13A	1.417(2)	1.412(3)	C32-C37
4	1.400(3)	C8A-C9A	1.394(3)	C8-C9	1.397(2)	1.417(4)	C21-C22	1.438(3)	C8-C9	1.437(3)	C8A-C9A	1.438(2)	1.416(3)	C32-C33
5	1.393(3)	C9A-C10A	1.395(3)	C9-C10	1.394(2)	1.396(5)	C20-C21	1.369(3)	C9-C10	1.370(3)	C9A-C10A	1.370(2)	1.380(3)	C33-C34
6	1.378(2)	C10A-C11A	1.378(3)	C10-C11	1.378(2)	1.390(5)	C19-C20	1.402(3)	C10-C11	1.400(3)	C10A-C11A	1.401(2)	1.401(3)	C34-C35
7	1.462(3)	C11A-C14A	1.459(2)	C11-C14	1.461(2)	1.446(4)	C16-C19	1.421(3)	C11-C14	1.418(3)	C11A-C14A	1.420(2)	1.438(3)	C35-C38
8	1.352(3)	C14A-C15A	1.362(3)	C14-C15	1.357(2)	1.412(4)	C14-C16	1.375(3)	C14-C15	1.377(3)	C14A-C15A	1.376(2)	1.374(3)	C15-C38
1’	1.487(2)	C12A-C15A	1.492(3)	C12-C15	1.490(2)	1.446(5)	C12-C14	1.464(3)	C12-C15	1.468(3)	C12A-C15A	1.466(2)	1.474(3)	C12-C15
2’	1.377(3)	C12A-C13A	1.382(2)	C12-C13	1.380(2)	1.400(4)	C12-C13	1.372(3)	C12-C13	1.371(3)	C12A-C13A	1.372(2)	1.375(3)	C12-C13
3’	1.408(2)	C8A-C13A	1.413(3)	C8-C13	1.411(2)	1.404(4)	C8-C13	1.416(3)	C8-C13	1.417(3)	C8A-C13A	1.417(2)	1.414(3)	C8-C13
4’	1.400(3)	C8A-C9A	1.394(3)	C8-C9	1.397(2)	1.402(4)	C8-C9	1.438(3)	C8-C9	1.437(3)	C8A-C9A	1.438(2)	1.423(3)	C8-C9
5’	1.393(3)	C9A-C10A	1.395(3)	C9-C10	1.394(2)	1.390(5)	C9-C10	1.369(3)	C9-C10	1.370(3)	C9A-C10A	1.370(2)	1.379(3)	C9-C10
6’	1.378(2)	C10A-C11A	1.378(3)	C10-C11	1.378(2)	1.395(5)	C10-C11	1.402(3)	C10-C11	1.400(3)	C10A-C11A	1.401(2)	1.401(3)	C10-C11
7’	1.462(3)	C11A-C14A	1.459(2)	C11-C14	1.461(2)	1.446(4)	C11-C15	1.421(3)	C11-C14	1.418(3)	C11A-C14A	1.420(2)	1.428(3)	C11-C14
8’	1.352(3)	C14A-C15A	1.362(3)	C14-C15	1.357(2)	1.398(4)	C15-C17	1.375(3)	C14-C15	1.377(3)	C14A-C15A	1.376(2)	1.374(3)	C14-C39
9	1.424(3)	C11A-C12A	1.423(3)	C11-C12	1.424(2)	1.437(4)	C18-C19	1.448(3)	C11-C12	1.451(3)	C11A-C12A	1.450(2)	1.435(3)	C35-C36
10	1.470(3)	C14A-C14A	1.464(3)	C14-C14	1.467(2)	1.434(4)	C15-C16	1.470(3)	C14-C14	1.467(3)	C14A-C14A	1.469(2)	1.472(3)	C14-C38
9’	1.424(3)	C11A-C12A	1.423(3)	C11-C12	1.424(2)	1.435(4)	C11-C12	1.448(3)	C11-C12	1.451(3)	C11A-C12A	1.450(2)	1.436(3)	C11-C12
11	1.480(3)	C5A-C8A	1.486(2)	C5-C8	1.483(2)	1.484(4)	C22-C24	1.440(3)	C5-C8	1.443(3)	C5A-C8A	1.442(2)	1.475(3)	C29-C32
12	1.371(2)	C2A-O1A	1.376(2)	C2-O1	1.374(2)	1.374(4)	C27-O2	1.327(3)	C2-O1	1.332(3)	C2A-O1A	1.330(2)	1.361(2)	C26-O2
11’	1.480(3)	C5A-C8A	1.486(2)	C5-C8	1.483(2)	1.485(4)	C5-C8	1.440(3)	C5-C8	1.443(3)	C5A-C8A	1.442(2)	1.460(3)	C5-C8
12’	1.371(2)	C2A-O1A	1.376(2)	C2-O1	1.374(2)	1.379(4)	C2-O1	1.327(3)	C2-O1	1.332(3)	C2A-O1A	1.330(2)	1.352(2)	C2-O1

Table S3. Bond length differences (in Å) of anion radical $\mathbf{1}^{\bullet-}$, dication $\mathbf{1}^{2+}$ and cation radical $\mathbf{1}^{\bullet+}$ compared to neutral **1**.

Bond #	anion radical ($\mathbf{1}^{\bullet-}$)*	dication ($\mathbf{1}^{2+}$)*,§	cation radical ($\mathbf{1}^{\bullet+}$)*
1	**−0.035(7)**	**−0.024(4)**	−0.019(5)
2	0.019(6)	−0.008(4)	0.002(5)
3	−0.019(6)	0.006(4)	0.002(5)
4	**0.020(6)**	**0.041(4)**	0.019(5)
5	0.002(7)	**−0.025(4)**	−0.014(5)
6	0.012(7)	**0.023(4)**	**0.023(5)**
7	−0.015(6)	**−0.041(4)**	**−0.023(5)**
8	**0.055(6)**	0.019(4)	0.017(5)
1’	**−0.044(7)**	**−0.024(4)**	−0.016(5)
2’	**0.021(6)**	−0.008(4)	−0.004(5)
3’	−0.006(6)	0.006(4)	0.004(5)
4’	0.005(6)	**0.041(4)**	**0.026(5)**
5’	−0.004(7)	**−0.025(4)**	−0.015(5)
6’	0.017(7)	**0.023(4)**	**0.023(5)**
7’	−0.015(6)	**−0.041(4)**	**−0.033(5)**
8’	**0.041(6)**	0.019(4)	0.017(5)
9	0.014(6)	**0.026(4)**	0.012(5)
10	**−0.033(6)**	0.002(4)	0.005(5)
9’	0.012(6)	**0.026(4)**	0.013(5)
11	0.001(6)	**−0.042(4)**	−0.008(5)
12	0.001(6)	**−0.044(4)**	−0.013(4)
11’	0.003(6)	**−0.042(4)**	**−0.023(5)**
12’	0.006(6)	**−0.044(4)**	**−0.021(4)**

*The arithmetic mean of **1** was used for calculation. §The arithmetic mean of $\mathbf{1}^{2+}$ was used for calculation. Values ≤ −0.020 Å and ≥ 0.020 Å are marked in bold.

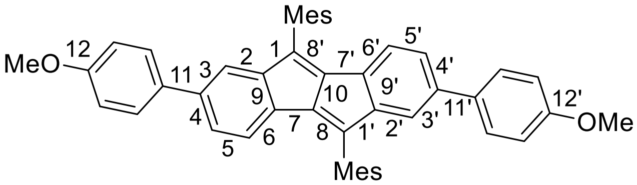

Figure S25. Numbering scheme for planar DPB **1** used in Bond length analysis in Table S2 and Table S3.

Table S4. Comparison of literature known **TIPS-DBP**.

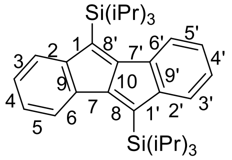

TIPS-DBP

CCDC<br>Lit.<br>#	neutral<br>622259<br>[11]<br>length / Å	anion radical<br>830385<br>[12]<br>length / Å	ΔL*/ Å	dianion<br>978684<br>[13]<br>length / Å	ΔL*/ Å
1	1.512(3)	1.436(8)	**−0.076(11)**	1.445(3)	**−0.067(6)**
2	1.376(4)	1.414(9)	**0.038(13)**	1.420(3)	**0.044(7)**
3	1.392(3)	1.379(9)	−0.013(12)	1.382(3)	−0.010(6)
4	1.371(4)	1.370(10)	−0.001(14)	1.401(2)	**0.030(6)**
5	1.398(4)	1.376(9)	**−0.022(13)**	1.381(3)	−0.017(7)
6	1.394(3)	1.378(8)	−0.016(11)	1.411(3)	0.017(6)
7	1.489(4)	1.433(8)	**−0.056(12)**	1.442(3)	**−0.047(7)**
8	1.358(4)	1.416(8)	**0.058(12)**	1.463(2)	**0.105(6)**
1’	1.512(3)	1.455(8)	**−0.057(11)**	1.445(3)	**−0.067(6)**
2’	1.376(4)	1.381(7)	0.005(11)	1.420(3)	**0.044(7)**
3’	1.392(3)	1.373(8)	−0.019(11)	1.382(3)	−0.010(6)
4’	1.371(4)	1.380(10)	0.009(14)	1.401(2)	**0.030(6)**
5’	1.398(4)	1.377(9)	**−0.021(13)**	1.381(3)	−0.017(7)
6’	1.394(3)	1.394(8)	0.000(11)	1.411(3)	0.017(6)
7’	1.489(4)	1.441(8)	**−0.048(12)**	1.442(3)	**−0.047(7)**
8’	1.358(4)	1.431(8)	**0.073(12)**	1.463(2)	**0.105(6)**
9	1.406(4)	1.460(10)	**0.054(14)**	1.463(2)	**0.057(6)**
10	1.466(3)	1.439(8)	**−0.027(11)**	1.433(2)	**−0.033(5)**
9’	1.406(4)	1.446(7)	**0.040(11)**	1.463(2)	**0.057(6)**

*Bond length difference compared to the neutral species. Values ≤ −0.020 Å and ≥ 0.020 Å are marked in bold.

5.2 Torsion angles

Table S5. Torsion angles (in °) of neutral **1,** anion radical $\mathbf{1}^{\bullet-}$, dication $\mathbf{1}^{2+}$ and cation radical $\mathbf{1}^{\bullet+}$.

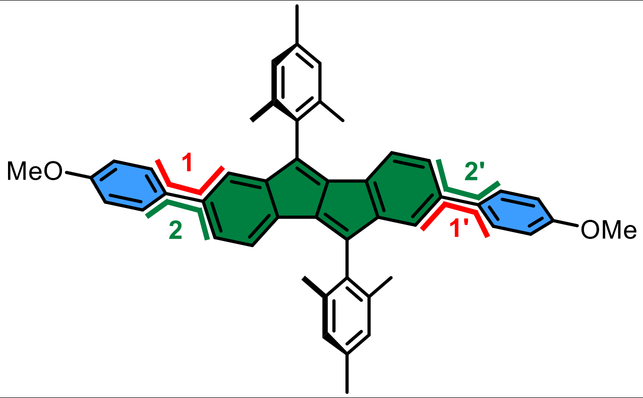

	neutral (**1**)*[1]			anion radical ($\mathbf{1}^{\bullet-}$)	dication ($\mathbf{1}^{2+}$)*			cation radical ($\mathbf{1}^{\bullet+}$)
#	A	B	average		A	B	average	
**1**	26.76	38.64	32.70	45.99	25.17	11.60	18.39	9.00
**1'**	§	§	§	45.57	§	§	§	9.46
**2**	28.02	37.51	32.77	44.17	27.43	12.05	19.74	9.79
**2'**	§	§	§	43.72	§	§	§	10.34
**Ø**	**27.39**	**38.08**	**32.73**	**44.86**	**26.30**	**11.83**	**19.06**	**9.65**

*Two molecules in the asymmetric unit, torsion angles of both molecules and their arithmetic mean are given. §Symmetry equivalent.

6 DFT Calculations

DFT calculations were performed with either the TURBOMOLE v7.4.1 program package[14] the Gaussian 16 program package[15] or the ORCA 4.2.1 program package.[16] The resolution-of-identity[17] (RI, RIJDX for SP) approximation for the Coulomb integrals was used in all DFT calculations employing matching auxiliary basis set def2-XVP/J.[18] Further, the D3 dispersion correction scheme[19,20] with the Becke-Johnson damping function was applied.[21,22] Using TURBOMOLE, the geometries the cations were optimized without symmetry restrictions with the PBEh-3c[23] composite scheme and the COSMO solvent model for chloroform, followed by harmonic vibrational frequency analysis to confirm minima as stationary points. Using Gaussian, the geometries of anions and neutral species were optimized without symmetry restrictions on the B3LYP/6-31G** level of theory with the PCM solvent model for tetrahydrofuran.[24] NICS values were calculated using the GIAO (Gauge Including Atomic Orbital)[25] method at the B3LYP/6-31G*[26] level of theory. NICS values were averaged over both 5- or 6-membered rings respectively. Calculation of EPR parameters was performed using ORCA on the U-B3LYP/EPR-II level of theory.[27]

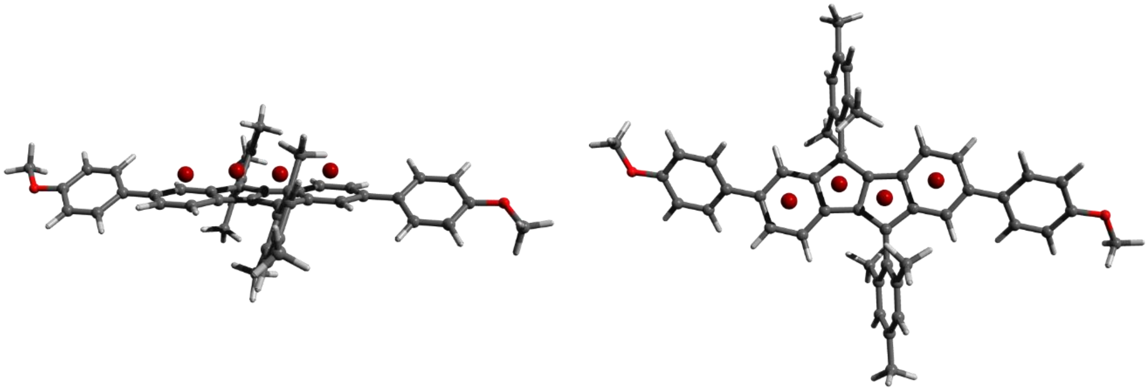

Figure S26. Exemplary position of the dummy atoms in $NICS(1)_{iso}$ calculations.

Table S6. $NICS(1)_{iso}$ values for **1** and its reduced and oxidized species.

	5-membered ring		6-membered ring	
Dication $\mathbf{1}^{2+}$	−3.20	−3.50	−6.40	−6.90
Cation radical $\mathbf{1}^{\bullet+}$	0.60	0.20	−5.70	−6.30
Neutral **1**	5.30	5.90	−6.20	−5.90
Anion radical $\mathbf{1}^{\bullet-}$	−2.70	−2.70	−7.50	−7.30
Dianion $\mathbf{1}^{2-}$	−9.20	−8.90	−9.10	−8.80

Table S7. Cartesian coordinates of **1**$^{2+}$ of the PBEh-3c level of theory.

C	-6.7375472	0.0110065	0.7042335
C	-5.7147221	0.7051713	0.0025146
C	-6.1269263	1.6779505	-0.949642
C	-7.4407373	1.9431867	-1.1735017
C	-8.4410535	1.2310371	-0.4640686
C	-8.0613744	0.2509568	0.4811617
C	-4.3271412	0.4338494	0.2424136
C	-3.3412094	0.8399074	-0.6969346
C	-2.0239343	0.5701586	-0.4497914
C	-1.6267725	-0.1169114	0.7356527
C	-2.5785571	-0.5160256	1.6663553
C	-3.9059378	-0.2438368	1.4140372
C	-0.8431882	0.855266	-1.2804577
C	0.2214318	0.3484855	-0.6050114
C	-0.2059012	-0.2569368	0.6552206
C	0.8588212	-0.7661707	1.3292583
C	2.0400483	-0.4773019	0.5003438
C	1.6419054	0.2050947	-0.687388
C	3.3582912	-0.7420203	0.7487963
C	4.3392843	-0.368308	-0.2089146
C	3.9155642	0.2932588	-1.3887613
C	2.5907023	0.5892545	-1.6271855
C	5.7253784	-0.6570834	0.0200307
C	6.2142284	-0.9709272	1.3183437
C	7.5279099	-1.2405028	1.5379916
C	8.4465749	-1.2312782	0.4576233
C	7.98762	-0.9314333	-0.845406
C	6.6686378	-0.6471267	-1.043319
O	9.6818311	-1.5094735	0.7578228
H	-3.6165589	1.3184324	-1.6271198
H	-4.6314503	-0.536874	2.1580403
H	-2.291479	-1.0335045	2.5717715
C	-0.8760346	1.538937	-2.5769131
C	0.8893803	-1.450774	2.6248981
H	2.304054	1.1211548	-2.5243677
H	4.6444592	0.6197118	-2.1153709
H	3.6308541	-1.2607131	1.6578496
H	-5.3945587	2.2578009	-1.4910364
H	-7.741591	2.7002678	-1.8846362
H	-8.8009678	-0.3185557	1.0251333
H	-6.4803722	-0.7608809	1.4139834
H	6.3447277	-0.4470076	-2.0536479
H	8.661135	-0.9313912	-1.6901732
H	5.5497954	-0.9630806	2.1693416
H	7.8919437	-1.4569863	2.5330078
O	-9.6720903	1.5439125	-0.7472663
C	-0.6550314	0.8102168	-3.7512419
C	-1.1358435	2.9168772	-2.6239999
C	10.7013928	-1.5288503	-0.2450179
C	-10.769034	0.8978077	-0.0955516
C	1.1680638	-2.821846	2.6738963
C	1.189634	-3.4527015	3.9127523
C	0.9542441	-2.7591405	5.0925566
C	0.6418907	-0.7252669	3.8002693
C	1.4102172	-3.6193507	1.4235261
H	1.3897026	-4.5173299	3.9539873
C	0.6840944	-1.3956627	5.0139689
C	0.3546824	0.7492824	3.7673405
C	0.9851271	-3.4515321	6.4221345
H	0.5045999	-0.8379097	5.9264614
H	0.7108468	-3.3596272	0.6282493
H	1.3054841	-4.6853604	1.6176908
H	2.4164405	-3.4645594	1.0293474
H	-0.6452194	0.9619005	3.3840816
H	1.0600743	1.2916501	3.1368673
H	0.4110806	1.1761795	4.7670687
H	1.2166289	-4.5103276	6.3199035
H	0.0242031	-3.368496	6.9315954
H	1.7343173	-3.008368	7.0794287
H	11.6196843	-1.783612	0.2722726
H	10.8062447	-0.5492501	-0.7107046
H	10.4884293	-2.2847614	-1.0005853
H	-11.664839	1.3452999	-0.5115374
H	-10.740202	1.0741094	0.9794328
H	-10.767815	-0.1721433	-0.3023817
C	-0.7052932	1.4811922	-4.9681717
C	-1.1664427	3.5450959	-3.860229
C	-0.9579406	2.8447524	-5.0449499
C	-0.3884661	-0.668138	-3.7202758
C	-1.3412209	3.7150013	-1.3676413
H	-1.352041	4.6127485	-3.9007555
H	-0.5456193	0.9196668	-5.8815468
C	-1.0044778	3.5545324	-6.3649203
H	-1.1235584	-1.2041957	-3.1186017
H	0.5939868	-0.8978679	-3.3036365
H	-0.4166014	-1.0874678	-4.7243598
H	-0.5724681	3.5065224	-0.6223977
H	-2.3042264	3.5044279	-0.8985557
H	-1.3148788	4.7827895	-1.5771298
H	-0.8183926	2.8748819	-7.1947166
H	-0.2585618	4.3490147	-6.4111338
H	-1.9773669	4.0201194	-6.5277007

Table S8. Cartesian coordinates of **1**$^{\bullet+}$ of the PBEh-3c level of theory.

C	-6.7412451	-0.0931029	0.5263922
C	-5.7400594	0.7439805	0.0282398
C	-6.143542	1.8729451	-0.7007546
C	-7.4723734	2.1510408	-0.9154106
C	-8.461788	1.2960468	-0.4153167
C	-8.0821304	0.164981	0.308586
C	-4.3263388	0.4562572	0.248245
C	-3.3613463	0.84929	-0.687211
C	-2.0327836	0.5805296	-0.4419892
C	-1.6302913	-0.1148857	0.7425552
C	-2.5847077	-0.5144663	1.6714288
C	-3.9081727	-0.2235352	1.4186588
C	-0.8640812	0.8619488	-1.2690379
C	0.2121383	0.3525259	-0.5989694
C	-0.2174069	-0.2588765	0.6663528
C	0.8546986	-0.7662142	1.3351628
C	2.0271716	-0.4769429	0.5096071
C	1.6204156	0.2129201	-0.6792372
C	3.3506133	-0.7589024	0.7451143

C	4.3161651	-0.3804409	-0.2022056
C	3.8944195	0.3002771	-1.3733843
C	2.5738178	0.601726	-1.6169835
C	5.726592	-0.6678466	0.0134321
C	6.2512798	-0.806998	1.3097382
C	7.5799932	-1.0820179	1.5198532
C	8.448465	-1.2458788	0.4324705
C	7.9476276	-1.1182221	-0.8649573
C	6.6114777	-0.827021	-1.0587215
O	9.7186985	-1.5170385	0.7282712
H	-3.6532198	1.3240502	-1.6152157
H	-4.6483065	-0.5055232	2.1548321
H	-2.3007354	-1.0388025	2.5740734
C	-0.8886759	1.5442333	-2.5647142
C	0.8924576	-1.4467322	2.6336218
H	2.290323	1.1448421	-2.5085047
H	4.6366614	0.6302424	-2.0865598
H	3.6341747	-1.2936504	1.6421163
H	-5.4055978	2.568827	-1.0784267
H	-7.7647047	3.0366773	-1.4640577
H	-8.8145748	-0.525971	0.7013189
H	-6.4797989	-0.9916597	1.0697301
H	6.2525484	-0.7607149	-2.0770455
H	8.5819078	-1.2492889	-1.7301428
H	5.6164981	-0.6616401	2.173596
H	7.9693105	-1.1661102	2.5258547
O	-9.7254007	1.639016	-0.675045
C	-0.5502122	0.8419894	-3.7293608
C	-1.2514565	2.8998604	-2.6282807
C	10.6537535	-1.6879181	-0.3216053
C	-10.775289	0.8220639	-0.1917596
C	1.217119	-2.8053773	2.698831
C	1.2589511	-3.423166	3.9456325
C	0.9974453	-2.7283855	5.1167258
C	0.6206241	-0.718635	3.8044367
C	1.5049707	-3.6220966	1.4701924
H	1.4969567	-4.4799058	3.9965859
C	0.6813798	-1.3746248	5.0237539
C	0.2961115	0.7478137	3.7597111
C	1.0495589	-3.405137	6.4539331
H	0.4820783	-0.8140306	5.9304384
H	1.0967005	-3.1811343	0.5623599
H	1.0812558	-4.6213456	1.5657878
H	2.5791799	-3.7453019	1.3189892
H	-0.6979078	0.9360307	3.3499277
H	1.00491	1.3050233	3.1460584
H	0.3166074	1.1777681	4.7596141
H	1.3125866	-4.4576516	6.3625369
H	0.0869942	-3.3454357	6.9637071
H	1.7860433	-2.9331128	7.1056373
H	11.6098548	-1.8925209	0.1513868
H	10.7468275	-0.7860448	-0.9306396
H	10.3903755	-2.5309554	-0.9642352
H	-11.700798	1.2908571	-0.5137945
H	-10.771975	0.7610365	0.8989653
H	-10.728081	-0.1869115	-0.6078922
C	-0.5817149	1.5155306	-4.9457804
C	-1.260483	3.5299094	-3.8635491
C	-0.9315454	2.8557855	-5.0355664
C	-0.174045	-0.6130868	-3.7117975
C	-1.599652	3.6918607	-1.3985911
H	-1.5237425	4.5810116	-3.9123776
H	-0.3326639	0.9712702	-5.8498393
C	-0.9571014	3.5679025	-6.3548427
H	-0.6485873	-1.1621803	-2.9002035
H	0.9047418	-0.7468315	-3.6051201
H	-0.4615389	-1.0937818	-4.6458292
H	-1.1082596	3.3171132	-0.5017734
H	-2.6744501	3.6832253	-1.2059064
H	-1.3084153	4.7343775	-1.5190831
H	-0.673398	2.9090589	-7.1737893
H	-0.27202	4.4167685	-6.3572286
H	-1.9518628	3.9594407	-6.5717261

Table S9. Cartesian coordinates of **1** on the B3LYP/6-31G** level of theory.

C	10.72412	0.32253	-0.56893
H	10.53622	0.30081	-1.64881
H	10.53984	-0.67389	-0.15049
H	11.76266	0.60222	-0.38981
O	9.9334	1.31515	0.07893
O	-9.93474	-1.31481	-0.07713
C	8.57556	1.20093	0.01548
C	6.45734	2.17148	0.67251
H	5.91495	2.94019	1.21428
C	7.84469	2.19943	0.67797
H	8.38622	2.98138	1.20059
C	7.89008	0.17747	-0.65006
H	8.42531	-0.60222	-1.17828
C	6.49417	0.16499	-0.64651
H	5.98285	-0.62362	-1.19016
C	5.74407	1.15329	0.00848
C	4.26076	1.12814	0.00262
C	3.51949	2.32077	-0.03486
H	4.04404	3.26953	-0.08752
C	2.11656	2.32123	-0.0413
H	1.57422	3.26091	-0.08326
C	1.44005	1.10914	-0.01238
C	2.17667	-0.11264	0.02768
C	3.56249	-0.10368	0.03244
H	4.11266	-1.03853	0.08195
C	1.22761	-1.26178	0.05056
C	0.0291	0.7326	-0.01874
C	-0.03027	-0.73334	0.01622
C	-1.22871	1.26112	-0.05262
C	-2.17785	0.11209	-0.02935
C	-1.44128	-1.10976	0.01039
C	-2.11794	-2.32177	0.03973
H	-1.57577	-3.26155	0.08165
C	-3.52088	-2.32115	0.03394
H	-4.04548	-3.26986	0.08697
C	-4.26206	-1.12847	-0.00331
C	-3.56366	0.10329	-0.03355
H	-4.11374	1.0382	-0.08285
C	-5.74537	-1.15345	-0.00852
C	-6.45905	-2.17174	-0.67195
H	-5.91699	-2.94068	-1.21374
C	-7.8464	-2.19952	-0.67681
H	-8.38825	-2.98156	-1.19897
C	-8.57687	-1.20074	-0.0143

C	-7.89098	-0.17717	0.65065
H	-8.42587	0.60275	1.17885
C	-6.49507	-0.16486	0.6465
H	-5.98343	0.62386	1.18969
C	-10.7250	-0.32188	0.57076
H	-10.5408	0.67438	0.15194
H	-10.5366	-0.29986	1.65056
H	-11.7637	-0.60149	0.39217
C	-1.63562	2.68888	-0.08777
C	-1.58594	3.46829	1.0892
C	-1.9902	4.80654	1.03445
H	-1.95837	5.40045	1.94523
C	-2.43744	5.39774	-0.15093
C	-2.48005	4.60737	-1.30363
H	-2.82548	5.0464	-2.237
C	-2.08881	3.26468	-1.29588
C	-1.11498	2.8798	2.40042
H	-1.26754	3.5872	3.21957
H	-0.04921	2.62834	2.36845
H	-1.65086	1.9565	2.64394
C	-2.83798	6.85367	-0.1913
H	-1.9817	7.4925	-0.44147
H	-3.22093	7.19008	0.77663
H	-3.60848	7.03587	-0.9463
C	-2.13145	2.46124	-2.57605
H	-2.47905	3.07593	-3.41013
H	-2.79978	1.59793	-2.49091
H	-1.14122	2.06941	-2.83298
C	1.63554	-2.68916	0.0868
C	1.57833	-3.47233	-1.08733
C	1.97795	-4.81187	-1.02967
H	1.93363	-5.41062	-1.93676
C	2.43276	-5.39936	0.15464
C	2.47513	-4.60752	1.30637
H	2.81361	-5.04725	2.24193
C	2.08866	-3.26349	1.29572
C	1.09721	-2.88889	-2.39713
H	0.0312	-2.63923	-2.35919
H	1.24589	-3.59849	-3.21509
H	1.6299	-1.96543	-2.64687
C	2.88913	-6.83902	0.18379
H	2.37195	-7.438	-0.57148
H	2.71192	-7.29498	1.16236
H	3.96441	-6.91719	-0.02015
C	2.12854	-2.45923	2.57546
H	2.79752	-1.59637	2.49091
H	2.47436	-3.07341	3.41066
H	1.13805	-2.06662	2.83018

Table S10. Cartesian coordinates of **1**$^{\bullet-}$ on the B3LYP/ 6-31G** level of theory.

C	-10.74212	-0.30736	-0.59315
H	-10.55267	-0.26447	-1.6727
H	-10.55811	0.68273	-0.15838
H	-11.78269	-0.58663	-0.42261
O	-9.95612	-1.31107	0.03782
O	9.95908	1.31089	-0.02924
C	-8.5929	-1.19057	-0.01998
C	-6.47051	-2.16071	0.62642
H	-5.92635	-2.93569	1.15773
C	-7.85911	-2.19601	0.62601
H	-8.39823	-2.99002	1.13374
C	-7.90861	-0.15524	-0.66624
H	-8.44481	0.63121	-1.18424
C	-6.51162	-0.13979	-0.66182
H	-6.00129	0.65716	-1.19416
C	-5.75322	-1.13107	-0.01805
C	-4.26993	-1.09846	-0.01396
C	-3.52331	-2.30139	-0.05461
H	-4.04836	-3.25009	-0.12188
C	-2.12713	-2.29403	-0.05842
H	-1.58371	-3.23446	-0.11332
C	-1.43213	-1.08084	-0.01403
C	-2.17943	0.16045	0.03453
C	-3.57868	0.12617	0.02609
H	-4.13805	1.05758	0.071
C	-1.25313	1.27988	0.05552
C	-0.03554	-0.72067	-0.01722
C	0.03824	0.72201	0.01351
C	1.2557	-1.27871	-0.05926
C	2.18214	-0.15946	-0.03708
C	1.43494	1.08195	0.01124
C	2.13012	2.295	0.05707
H	1.58686	3.23551	0.11209
C	3.52631	2.30213	0.05493
H	4.05142	3.25072	0.12339
C	4.27279	1.09912	0.01463
C	3.58139	-0.12537	-0.0269
H	4.14069	-1.05685	-0.07129
C	5.75609	1.13149	0.02076
C	6.47441	2.16135	-0.62221
H	5.93109	2.93669	-1.15386
C	7.86301	2.19645	-0.61987
H	8.40294	2.99064	-1.12645
C	8.59576	1.19057	0.02661
C	7.91043	0.15501	0.67141
H	8.44579	-0.63178	1.18975
C	6.51345	0.13977	0.66506
H	6.00229	-0.65737	1.19631
C	10.74405	0.30675	0.60235
H	10.56053	-0.68309	0.16681
H	10.55307	0.26334	1.6816
H	11.7849	0.58597	0.43342
C	1.63385	-2.71221	-0.09218
C	1.41598	-3.54161	1.03605
C	1.78555	-4.89034	0.98685
H	1.62058	-5.51175	1.86539
C	2.37153	-5.45848	-0.1486
C	2.58422	-4.6304	-1.25421
H	3.03349	-5.05033	-2.15251
C	2.22444	-3.27746	-1.24911
C	0.81278	-2.98453	2.30569
H	0.86085	-3.71956	3.11471
H	-0.23619	-2.70375	2.16404
H	1.33641	-2.07897	2.62919
C	2.73421	-6.92515	-0.18857
H	1.89204	-7.53713	-0.53687
H	3.01207	-7.29621	0.80304
H	3.57134	-7.11276	-0.86813
C	2.44287	-2.44869	-2.49511

H	2.73297	-3.08154	-3.33902
H	3.22506	-1.69528	-2.35319
H	1.53352	-1.90299	-2.76717
C	-1.63335	2.71278	0.08863
C	-1.41276	3.54424	-1.03758
C	-1.78052	4.89335	-0.98606
H	-1.60715	5.51782	-1.86079
C	-2.37207	5.45877	0.14794
C	-2.57972	4.6307	1.25449
H	-3.02127	5.0516	2.15615
C	-2.2216	3.27731	1.24712
C	-0.80185	2.99072	-2.30511
H	0.24627	2.7094	-2.15859
H	-0.84576	3.72772	-3.11254
H	-1.32384	2.08603	-2.63373
C	-2.79436	6.90983	0.16646
H	-2.13129	7.52838	-0.44668
H	-2.7918	7.31456	1.18327
H	-3.81033	7.03696	-0.22952
C	-2.43245	2.44897	2.4947
H	-3.21495	1.69511	2.3568
H	-2.71898	3.0819	3.33976
H	-1.5215	1.90381	2.76245

Table S11. Cartesian coordinates of **1**$^{2-}$ on the B3LYP/6-31G** level of theory.

C	-10.7865	-0.21421	-0.39135
H	-10.59518	0.13238	-1.41604
H	-10.59907	0.6191	0.29943
H	-11.8321	-0.51749	-0.30814
O	-10.01186	-1.35426	-0.06565
O	10.01366	1.35315	0.08419
C	-8.63561	-1.21514	-0.08502
C	-6.50696	-2.32165	0.28294
H	-5.97789	-3.217	0.59478
C	-7.89192	-2.35749	0.26362
H	-8.42645	-3.26422	0.53505
C	-7.94593	-0.04379	-0.41507
H	-8.48167	0.85651	-0.69766
C	-6.54995	-0.01961	-0.39889
H	-6.05673	0.90026	-0.69547
C	-5.76394	-1.15301	-0.0517
C	-4.30311	-1.11705	-0.02666
C	-3.52895	-2.30969	-0.06657
H	-4.0355	-3.26843	-0.14789
C	-2.13734	-2.30378	-0.06197
H	-1.59822	-3.24711	-0.1251
C	-1.42673	-1.08376	-0.00807
C	-2.19034	0.15293	0.03936
C	-3.58171	0.1227	0.01839
H	-4.12786	1.0616	0.05384
C	-1.2555	1.27881	0.05355
C	-0.0422	-0.72596	-0.01425
C	0.04344	0.72704	0.00667
C	1.25673	-1.27774	-0.0615
C	2.19163	-0.15199	-0.04545
C	1.42806	1.08471	0.00181
C	2.13891	2.3045	0.0585
H	1.59991	3.2479	0.12166
C	3.53047	2.31012	0.06651
H	4.03675	3.26878	0.15011
C	4.30473	1.11744	0.02702
C	3.58297	-0.12212	-0.02161
H	4.12869	-1.06126	-0.0563
C	5.76541	1.15302	0.05637
C	6.50989	2.32338	-0.26928
H	5.98222	3.22082	-0.57741
C	7.89477	2.35876	-0.24551
H	8.43027	3.26695	-0.51011
C	8.63725	1.21426	0.09876
C	7.94624	0.04127	0.42003
H	8.48089	-0.86079	0.69912
C	6.55033	0.01746	0.39944
H	6.05626	-0.90406	0.68932
C	10.78699	0.21098	0.40545
H	10.60119	-0.61828	-0.29064
H	10.59273	-0.14146	1.42759
H	11.83293	0.51434	0.32689
C	1.63737	-2.70664	-0.07873
C	1.34612	-3.54994	1.02775
C	1.72241	-4.89604	0.99807
H	1.50034	-5.52128	1.86197
C	2.39279	-5.45808	-0.09487
C	2.67817	-4.62249	-1.17739
H	3.18884	-5.03689	-2.04554
C	2.31104	-3.27072	-1.19308
C	0.66608	-3.00162	2.26131
H	0.63652	-3.75323	3.05661
H	-0.35972	-2.68563	2.04732
H	1.18984	-2.11693	2.63982
C	2.77024	-6.92173	-0.10789
H	1.90412	-7.56231	-0.32019
H	3.17139	-7.24214	0.86022
H	3.52553	-7.13292	-0.87111
C	2.60084	-2.44259	-2.42357
H	2.93515	-3.07507	-3.252
H	3.37132	-1.68645	-2.23574
H	1.70755	-1.89424	-2.74054
C	-1.63711	2.70747	0.07052
C	-1.34295	3.55224	-1.03284
C	-1.71593	4.90013	-0.9997
H	-1.48538	5.52839	-1.85898
C	-2.38937	5.45996	0.09133
C	-2.6718	4.62355	1.17518
H	-3.17521	5.03873	2.04729
C	-2.3079	3.2718	1.1877
C	-0.6572	3.00801	-2.26509
H	0.36937	2.69549	-2.04958
H	-0.62872	3.76062	-3.0595
H	-1.177	2.12196	-2.64581
C	-2.81873	6.90929	0.08572
H	-2.12241	7.53241	-0.48509
H	-2.87868	7.31362	1.10143
H	-3.80995	7.03639	-0.36994
C	-2.59034	2.44374	2.41998
H	-3.35935	1.68546	2.2351
H	-2.92373	3.07582	3.24907
H	-1.69439	1.89818	2.73424

7 References

[1] M. Hermann, D. Wassy, D. Kratzert, B. Esser, *Chem. – A Eur. J.* **2018**, *24*, 7374–7387.

[2] M. Schorpp, T. Heizmann, M. Schmucker, S. Rein, S. Weber, I. Krossing, *Angew. Chem.* **2020**, *132*, 9540–9546.

[3] G. R. Fulmer, A. J. M. Miller, N. H. Sherden, H. E. Gottlieb, A. Nudelman, B. M. Stoltz, J. E. Bercaw, K. I. Goldberg, *Organometallics* **2010**, *29*, 2176–2179.

[4] K. Herb, R. Tschaggelar, G. Denninger, G. Jeschke, *J. Magn. Reson.* **2018**, *289*, 100–106.

[5] S. Stoll, A. Schweiger, *J. Magn. Reson.* **2006**, *178*, 42–55.

[6] J. Cosier, A. M. Glazer, *J. Appl. Crystallogr.* **1986**, *19*, 105–107.

[7] G. M. Sheldrick, *Acta Crystallogr. Sect. C Struct. Chem.* **2015**, *71*, 3–8.

[8] D. Kratzert, J. J. Holstein, I. Krossing, *J. Appl. Crystallogr.* **2015**, *48*, 933–938.

[9] D. Kratzert, I. Krossing, *J. Appl. Crystallogr.* **2018**, *51*, 928–934.

[10] D. Kratzert, FinalCif, https://www.xs3.uni-freiburg.de/research/finalcif, **2020**.

[11] M. Saito, M. Nakamura, T. Tajima, M. Yoshioka, *Angew. Chem. Int. Ed.* **2007**, *46*, 1504–1507.

[12] M. Saito, Y. Hashimoto, T. Tajima, K. Ishimura, S. Nagase, M. Minoura, *Chem. - An Asian J.* **2012**, *7*, 480–483.

[13] T. Kuwabara, K. Ishimura, T. Sasamori, N. Tokitoh, M. Saito, *Chem. - A Eur. J.* **2014**, *20*, 7571–7575.

[14] TURBOMOLE V7.4.1, a development of the University of Karlsruhe and Forschungszentrum Karlsruhe GmbH, **2019**.

[15] Gaussian 16, Revision C.01, M. J. Frisch, G. W. Trucks, H. B. Schlegel, G. E. Scuseria, M. A. Robb, J. R. Cheeseman, G. Scalmani, V. Barone, G. A. Petersson, H. Nakatsuji, X. Li, M. Caricato, A. V. Marenich, J. Bloino, B. G. Janesko, R. Gomperts, B. Mennucci, H. P. Hratchian, J. V. Ortiz, A. F. Izmaylov, J. L. Sonnenberg, D. Williams-Young, F. Ding, F. Lipparini, F. Egidi, J. Goings, B. Peng, A. Petrone, T. Henderson, D. Ranasinghe, V. G. Zakrzewski, J. Gao, N. Rega, G. Zheng, W. Liang, M. Hada, M. Ehara, K. Toyota, R. Fukuda, J. Hasegawa, M. Ishida, T. Nakajima, Y. Honda, O. Kitao, H. Nakai, T. Vreven, K. Throssell, J. A. Montgomery, Jr., J. E. Peralta, F. Ogliaro, M. J. Bearpark, J. J. Heyd, E. N. Brothers, K. N. Kudin, V. N. Staroverov, T. A. Keith, R. Kobayashi, J. Normand, K. Raghavachari, A. P. Rendell, J. C. Burant, S. S. Iyengar, J. Tomasi, M. Cossi, J. M. Millam, M. Klene, C. Adamo, R. Cammi, J. W. Ochterski, R. L. Martin, K. Morokuma, O. Farkas, J. B. Foresman, and D. J. Fox, Gaussian, Inc., Wallingford CT, **2016**.

[16] F. Neese, *Wiley Interdiscip. Rev. Comput. Mol. Sci.* **2018**, *8*, DOI 10.1002/wcms.1327.

[17] K. Eichkorn, O. Treutler, H. Öhm, M. Häser, R. Ahlrichs, *Chem. Phys. Lett.* **1995**, *240*, 283–290.

[18] F. Weigend, *Phys. Chem. Chem. Phys.* **2006**, *8*, 1057–1065.

[19] S. Grimme, J. Antony, S. Ehrlich, H. Krieg, *J. Chem. Phys.* **2010**, *132*, 154104.

[20] S. Grimme, S. Ehrlich, L. Goerigk, *J. Comput. Chem.* **2011**, *32*, 1456–1465.

[21] A. D. Becke, E. R. Johnson, *J. Chem. Phys.* **2005**, *123*, 154101.

[22] E. R. Johnson, A. D. Becke, *J. Chem. Phys.* **2006**, *124*, 174104.

[23] S. Grimme, J. G. Brandenburg, C. Bannwarth, A. Hansen, *J. Chem. Phys.* **2015**, *143*, 054107.

[24] S. Miertuš, E. Scrocco, J. Tomasi, *Chem. Phys.* **1981**, *55*, 117–129.

[25] M. Häser, R. Ahlrichs, H. P. Baron, P. Weis, H. Horn, *Theor. Chim. Acta* **1992**, *83*, 455–470.

[26] G. A. Petersson, A. Bennett, T. G. Tensfeldt, M. A. Al-Laham, W. A. Shirley, J. Mantzaris, *J. Chem. Phys.* **1988**, *89*, 2193–2218.

[27] V. BARONE, in *Recent Advances in Density Functional Methods (Part I)* (Ed.: D.P. Chong), World Scientific Publishing Co. Pte. Ltd., Singapore, DOI: 10.1142/9789812830586_0008, **1995**, pp. 287–334.
